# On probability measures arising from lattice points on circles

**DOI:** 10.1007/s00208-016-1411-4

**Published:** 2016-04-23

**Authors:** Pär Kurlberg, Igor Wigman

**Affiliations:** 1 Department of Mathematics, KTH Royal Institute of Technology, 100 44 Stockholm, Sweden; 2 Department of Mathematics, King’s College London, London, UK

## Abstract

A circle, centered at the origin and with radius chosen so that it has non-empty intersection with the integer lattice ${\mathbb Z}^{2}$, gives rise to a probability measure on the unit circle in a natural way. Such measures, and their weak limits, are said to be *attainable* from lattice points on circles. We investigate the set of attainable measures and show that it contains all extreme points, in the sense of convex geometry, of the set of all probability measures that are invariant under some natural symmetries. Further, the set of attainable measures is closed under convolution, yet there exist symmetric probability measures that are *not* attainable. To show this, we study the geometry of projections onto a finite number of Fourier coefficients and find that the set of attainable measures has many singularities with a “fractal” structure. This complicated structure in some sense arises from prime powers—singularities do not occur for circles of radius $\sqrt{n}$ if *n* is *square free*.

## Introduction

Let *S* be the set of nonzero integers expressible as a sum of two integer squares. For $n\in S$, let

$$\begin{aligned} \Lambda _{n} := \{ \vec {\lambda }= a+bi \in {\mathbb Z}[i]:\, a^{2}+b^{2}=n \} \end{aligned}$$

denote the intersection of the lattice ${\mathbb Z}[i] \subset \mathbb {C}$ with a circle centered at the origin and of radius $\sqrt{n}$. For $n \in S$, let $r_{2}(n) := |\Lambda _{n} |$ denote the cardinality of $\Lambda _{n}$; for $n \not \in S$ it is convenient to define $r_{2}(n)=0$. We define a probability measure $\mu _{n}$ on the unit circle

$$\begin{aligned} \mathcal {S}^{1} := \{ z \in \mathbb {C}: |z|=1 \} \end{aligned}$$

by letting

$$\begin{aligned} \mu _{n} := \frac{1}{r_{2}(n)}\sum \limits _{\vec {\lambda }\in \Lambda _{n}}\delta _{\vec {\lambda }/\sqrt{n}}, \end{aligned}$$

where $\delta _{z}$ denotes the Dirac delta function with support at *z*. The measures $\mu _{n}$ are clearly invariant under multiplication by *i* and under complex conjugation. We say that a measure on $\mathcal {S}^{1}$ is *symmetric* if it is invariant under these symmetries.

### **Definition 1.1**

A probability measure $\nu$ is said to be **attainable from lattice points on circles**, or simply just **attainable**, if $\nu$ is a weak limit point of the set $\{\mu _{n}\}_{n \in S}$.

We note that any attainable measure is automatically symmetric. Now, if two integers $m,n \in S$ are co-prime,

1 $$\begin{aligned} \mu _{mn} = \mu _m \bigstar \mu _n, \end{aligned}$$

where $\bigstar$ denotes convolution of measures on $\mathcal {S}^{1}$. Thus measures $\mu _{n}$ for *n* a prime power are of particular interest. It turns out that the closure of the set of measures given by $\mu _{p^e}$ for *p* ranging over all primes $p \equiv 1 \mod 4$ and exponents *e* ranging over integers $e \ge 1$ contains $\mu _{2^{k}}$, as well as $\mu _{q^{2k}}$ for any prime $q \equiv 3 \mod 4$, and any exponent $k \ge 0$. (Note that $q^{l} \in S$ forces *l* to be even.)

Motivated by the above, we say that a measure $\mu$ is *prime power attainable* of $\mu$ is a weak limit point of the set $\{ \mu _{p^{e}} \}_{p \equiv 1 \text { mod } 4, \, e \ge 1}$. Similarly, we say that a measure $\mu$ is *prime attainable* if $\mu$ is a weak limit point of the set $\{ \mu _{p} \}_{p \equiv 1 \text { mod } 4}$.

### **Proposition 1.2**

The set of attainable measures is closed under convolution. Further, it is the closure (in the weak topology) of the collection of all convolutions of finitely many prime power attainable measures, i.e., it is topologically generated by the prime power attainable measures.

**Fig. 1:**
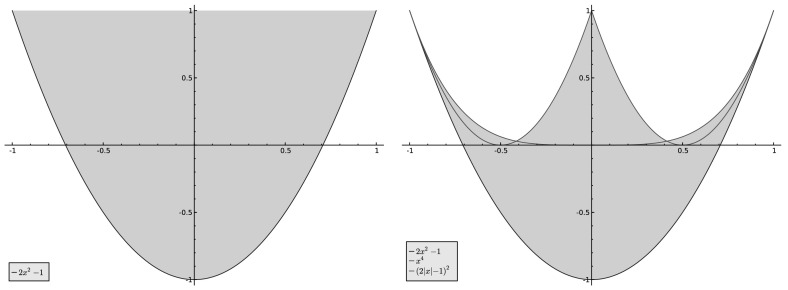
*Left*: $\{ (\hat{\mu }(4), \hat{\mu }(8)) : \mu \text { is symmetric}\}$. *Right*: the region defined by the inequalities $2x^{2}-1 \le y \le \max (x^{4}, (2|x|-1)^{2} )$

Hence the set of attainable measures is the smallest closed (in the weak topology) set containing all the prime power attainable measures and closed w.r.t. convolution of probability measures. The set of all symmetric probability measures is clearly a convex set, hence equals the convex hull of its extreme points. Quite interestingly, the set of prime attainable measures is exactly the set of extreme points. Now, since the set of attainable measures contains the extreme points, and is closed under convolution one might wonder if *all* symmetric probability measures are attainable? By studying Fourier coefficients of attainable measures we shall show that **not all symmetric measures are attainable**.

Given a measure $\mu$ on $\mathcal {S}^{1}$ and $k \in {\mathbb Z}$, define the *k*-th Fourier coefficient of $\mu$ by

$$\begin{aligned} \hat{\mu }(k) := \int _{\mathcal {S}^{1}} z^{-k} d\mu (z). \end{aligned}$$

If $\mu$ is symmetric it is straightforward to see that $\hat{\mu }(k) = 0$ unless 4|*k*. Since $\mu$ is a probability measure, $\hat{\mu }(0) = 1$, hence the first two informative Fourier coefficients are $\hat{\mu }(4)$ and $\hat{\mu }(8)$; note that $\hat{\mu }(-k) = \hat{\mu }(k)$ for all *k* since $\mu$ is both real and even (i.e. it is invariant under complex conjugation).

### **Theorem 1.3**

If $\mu$ is attainable and $|\hat{\mu }(4)|> 1/3$ then

2 $$\begin{aligned} 2\hat{\mu }(4)^{2}-1 \le \hat{\mu }(8) \le \mathcal {M}(\hat{\mu }(4)), \end{aligned}$$

where

3 $$\begin{aligned} \mathcal {M}(x) = \max \left( x^{4}, (2|x|-1)^{2} \right) \end{aligned}$$

denotes the “max curve”. Conversely, given *x*, *y* such that $|x| \le 1$ and

$$\begin{aligned} 2x^{2}-1 \le y \le \mathcal {M}(x) , \end{aligned}$$

there exists an attainable measure $\mu$ such that $( \hat{\mu }(4), \hat{\mu }(8)) = (x,y)$.

For comparison, we note that the Fourier coefficients of the full set of symmetric probability measures has the following quite simple description (see Sect. 3.2 below):

$$\begin{aligned} \{ (\hat{\mu }(4), \hat{\mu }(8)) : \mu \text { is symmetric} \} = \{ (x,y) : |x| \le 1, \ 2x^{2}-1 \le y \le 1 \}. \end{aligned}$$

As Fig. 1 illustrates, the discrepancy between all symmetric measures and the attainable ones is fairly large. In particular, note that the curves $y=x^{4}$, $y = 2x^{2}-1$, and $(2|x|-1)^{2}$ all have the *same tangent* at the two points $(\pm 1, 1)$, consequently the set of attainable measures has cusps near $(\pm 1, 1)$. However, there are attainable measures corresponding to points *above the red curve* for $|x| \le 1/3$.

**Fig. 2:**
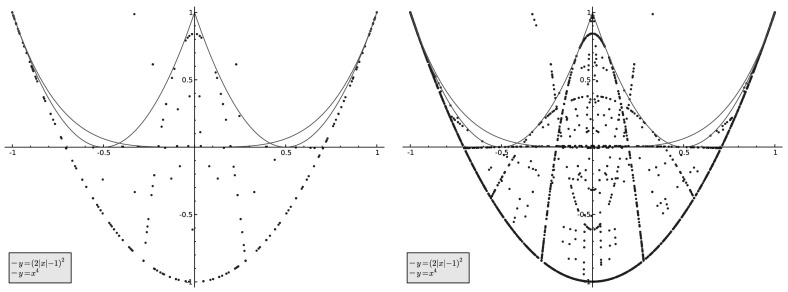
*Left*: $(\hat{\mu _n}(4), \hat{\mu _n}(8))$ for $n\in S$, $n\le 1000$. *Right*: $(\hat{\mu _n}(4), \hat{\mu _n}(8))$ for $n \in S, n \le 10000$

**Fig. 3:**
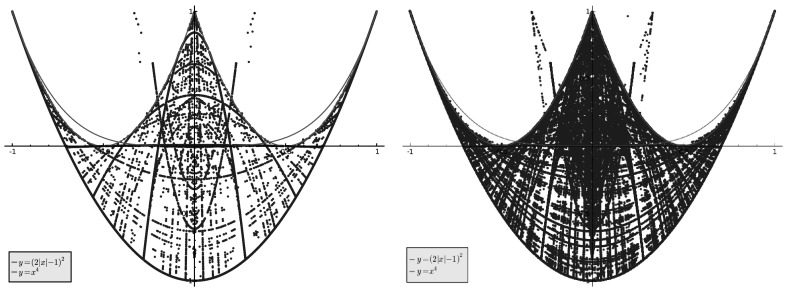
*Left*: $(\hat{\mu _n}(4), \hat{\mu _n}(8))$ for $n \in S, n \le 100000$. *Right*: $(\hat{\mu _n}(4), \hat{\mu _n}(8))$ for $n \in S, n \le 1000000$

To give an indication of the rate at which the admissible region is “filled out”, as well as illuminate what happens in the region $|\hat{\mu }(4)| \le 1/3$, we next present the results of some numerical experiments in Figs. 2 and 3.

Note that points lying clearly above the red curve, but below the green one, are quite rare. However, “spikes” in the region $|\hat{\mu }(n)| \le 1/3$ are clearly present.

### Square free attainable measures

As we shall see, the spikes in the region $|\hat{\mu }(4)| \le 1/3$ are limits of measures $\mu _n$ where *n* is divisible by $p^{e}$ for $e \ge 2$, but for measures arising from square free $n \in S$, the structure is much simpler.

We say that a measure $\mu$ is *square free attainable* if $\mu$ is a limit point of the set $\{ \mu _{n} : n \in S \text { and n is square free}\}$. The set of square free attainable measures is also closed under convolution, and it is easy to see that it is generated by the set $\{\mu _p\}_{p \equiv 1 \mod 4}$, whose closure is the set of prime attainable measures.

#### **Theorem 1.4**

If $\mu$ is square free attainable then

4 $$\begin{aligned} 2\hat{\mu }(4)^{2}-1 \le \hat{\mu }(8) \le \mathcal {M}(\hat{\mu }(4)). \end{aligned}$$

Conversely, if $2x^{2}-1 \le y \le \mathcal {M}(x)$ there exists a square free attainable measure $\mu$ such that $(\hat{\mu }(4), \hat{\mu }(8)) = (x,y)$.

The proof of Theorem 1.4 is very similar to the proof of Proposition 1.2, cf. Remark 4.2.

### Prime power attainable measures

**Fig. 4:**
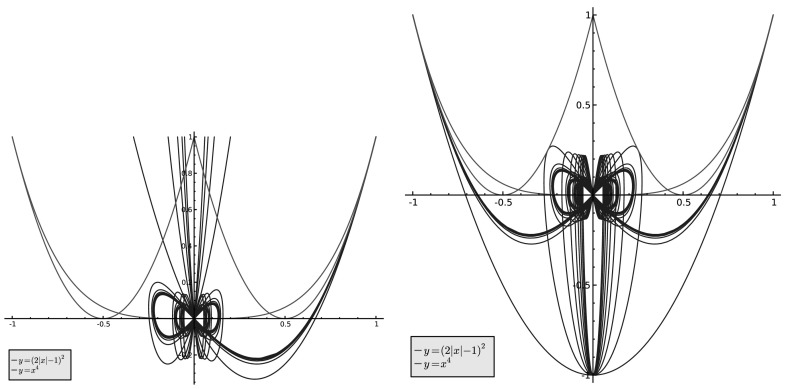
Prime power attainable measures attainable by $p^{M}$, $p\equiv 1(4)$ primes, $M\le 19$. *Left picture*: even *M*. *Right picture*: odd *M*

As mentioned before, the spikes in the region $|\hat{\mu }(4)| \le 1/3$ are due to measures $\mu _n$ for which *n* is divisible by a prime power $p^{e}$, for *e* large. Recall that a measure $\mu$ is prime power attainable if $\mu$ is a weak limit point of the set $\{\mu _{p^{e}}\}_{p \equiv 1 \mod 4, e \ge 1}$. If $\mu$ is a prime power attainable measure, then the point $(\hat{\mu }(4), \hat{\mu }(8))$ can indeed lie *above* the curve $\max ( x^{4}, (2|x|-1)^{2})$ in the region $|\hat{\mu }(4)| \le 1/3$, though this phenomenon only occurs for even exponents (see Fig. 4).

In fact, we will show that for every $k \in {\mathbb Z}^+$ there exists prime power attainable $\mu$ such that

$$\begin{aligned} (\hat{\mu }(4), \hat{\mu }(8)) = \left( \frac{1}{2k+1}, 1\right) . \end{aligned}$$

### Fractal structure for $|\hat{\mu }(4)| \le \frac{1}{3}$

**Fig. 5:**
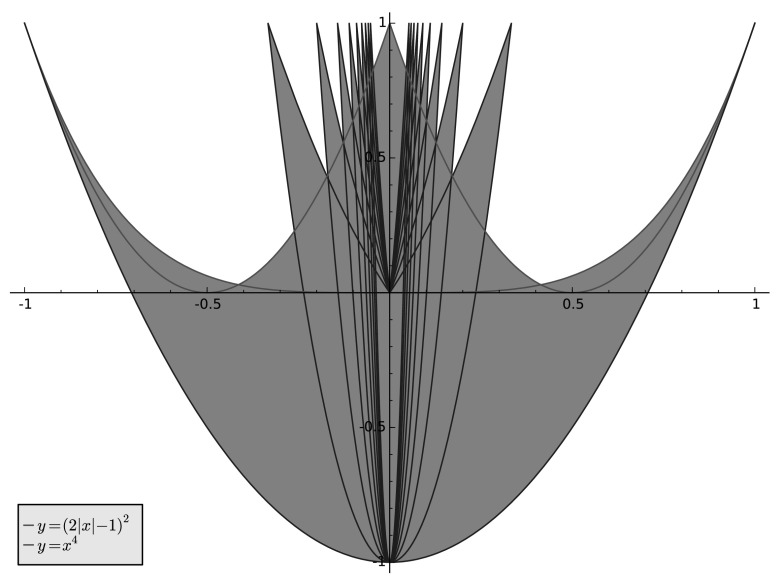
Points $(\hat{\mu }(4), \hat{\mu }(8))$ for some attainable measures $\mu$ giving rise to spikes in the region $|\hat{\mu }(4)| \le 1/3$

Let

5 $$\begin{aligned} \mathcal {A}_{2} := \{ (\hat{\mu }(4), \hat{\mu }(8)) : \mu \,\, \text {is attainable} \} \end{aligned}$$

denote the projection of the set of attainable measures onto the first two non-trivial Fourier coefficients. The intersection of $\mathcal {A}_{2}$ with the vertical strip $\{ (x,y) : |x| \le 1/3\}$ turns out to have a rather complicated fractal structure with infinitely many spikes—see Fig. 5. Since $\mathcal {A}_{2}$ is closed under multiplication and $(-1,1)\in \mathcal {A}_{2}$ it implies that it is invariant w.r.t.

6 $$\begin{aligned} (x,y)\mapsto (-x,y), \end{aligned}$$

and hence we may assume $x\ge 0$.

To be able to give a complete description of $\mathcal {A}_{2}$ we need a definition.

#### **Definition 1.5**

Let $$x_{0}\in [0,1]$$ and $$a < x_{0}$$.

1. We say that a pair of continuous functions
  $$\begin{aligned} f_{1},f_{2}:(a,x_{0}]\rightarrow [0,1], \end{aligned}$$
  defines a **cornered domain between** *a*  **and**  $x_{0}$ if for all $x\in (a,x_{0}]$ one has $f_{1}(x)\le f_{2}(x)$, and $f_{1}(x)=f_{2}(x)$ if and only if $x=x_{0}$, whence $f_{1}(x_{0})=f_{2}(x_{0})=1$.
2. For a pair of functions $f_{1},f_{2}$ as above the corresponding **cornered domain between**  *a*  **and**  $x_{0}$ is
  $$\begin{aligned} \mathcal {D}_{a,x_{0}}(f_{1},f_{2}) = \{ (x,y)\in {\mathbb R}^{2}:\, x\in (a,x_{0}],\, f_{1}(x) \le y\le f_{2}(x) \}. \end{aligned}$$

The functions $f_{1}$ and $f_{2}$ will be referred to as the “lower and upper” bounds for $\mathcal {D}_{a,x_{0}}(f_{1},f_{2})$ respectively.

#### **Theorem 1.6**

The intersection of the set $\mathcal {A}_{2}$ with the line $y=1$ equals

$$\begin{aligned} \left\{ \left( \frac{\pm 1}{2k+1},1\right) :\, k\ge 1 \right\} \cup \{ (0,1)\} \cup \{ (\pm 1,1)\}. \end{aligned}$$

Further, for $k\ge 1$, let $x_{k}=\frac{1}{2k+1}$ be the *x*-coordinate of a point of the intersection described above. Then, for every $k\ge 1$ there exists a pair of continuous piecewise analytic functions $f_{1;k},\,f_{2;k}$ defining a cornered domain between 0 and $x_{k}$, so that $\mathcal {A}_{2}$ admits the following global description:

7 $$\begin{aligned}&\mathcal {A}_{2}\cap \left\{ 0<x \le \frac{1}{3} \right\} = \left( \bigcup \limits _{k=1}^{\infty } \mathcal {D}_{0,x_{k}}(f_{1;k},f_{2;k})\right) \nonumber \\&\quad \bigcup \left\{ (x,y):0<x\le \frac{1}{3},\,2x^{2}-1\le y\le (2x-1)^{2} \right\} . \end{aligned}$$

Theorem 1.6 is a rigorous explanation of the thin strips or “spikes” connecting all the reciprocals of odd numbers on $y=1$, and the curve $y=(2|x|-1)^{2}$, as in Fig. 5. We remark that the functions $f_{1;k}$ and $f_{2;k}$ can with some effort be computed explicitly. The lower bound $f_{1;k}$ is given as the (component-wise) product of $(x_{k},1)$ by the parabola $y=2x^{2}-1$ mapping $(1,1)\mapsto (x_{k},1)$; we re-parameterize the resulting curve $(x \cdot x_{k},2x^{2}-1)$ so that it corresponds to the function

8 $$\begin{aligned} f_{1;k}(x) = \frac{2}{x_{k}^{2}} x^{2}-1, \end{aligned}$$

whose slope at $x_{k}$ is $f_{1;k}'(x_{k}) = 4(2k+1)$.

The upper bound $f_{2;k}(x)$ is of a somewhat more complicated nature, see Definition 6.3; it is analytic around the corner with the slope $f_{2;k}'(x_{k}) = \frac{4}{3}(2k+1)$ (see the proof of Theorem 1.6 in Sect. 6), and it is *plausible* that it is (everywhere) analytic. It then follows that the set $\mathcal {A}_{2}$ has a discontinuity, or a jump, at $x=x_{k}$ (this is a by-product of the fact that the slopes of both $f_{1;k}$ and $f_{2;k}$ at $x_{k}$ are *positive*.)

### Discussion

Our interest in attainable measures originates in the study [5] of zero sets (“nodal lines”) of random Laplace eigenfunctions on the standard torus ${\mathbb T}:= {\mathbb R}^2/{\mathbb Z}^2$. More precisely, for each $n \in S$ there is an associated Laplace eigenvalue given by $4\pi ^2 n$, with eigenspace dimension equal to $r_{2}(n)$. On each such eigenspace there is a natural notion of a “random eigenfunction”, and the variance (appropriately normalized) of the nodal line lengths of these random eigenfunctions equals $(1+\widehat{\mu _{n}}(4)^{2})/512 + o(1)$ as $r_{2}(n) \rightarrow \infty$. It was thus of particular interest to show that the accumulation points of $\widehat{\mu _{n}}(4)^{2}$, as $n \in S$ tends to infinity in such a way that also the eigenspace dimension $r_{2}(n) \rightarrow \infty$, is maximal—namely the full interval [0, 1]. This is indeed the case (cf. [5, Section 1.4]), but a very natural question is: which measures are attainable?

In order to obtain asymptotics for the above variance it is essential to assume that the eigenspace dimension grows, and one might wonder if “fewer” measures are attainable under this additional assumption. However, as the following shows, this is not the case (the proof can be found in Sect. 4.4.)

#### **Proposition 1.7**

A measure $\mu \in \mathcal {P}$ is attainable (i.e. $\mu \in \mathcal {A}$), if and only if there exists a sequence $\{n_{j}\}$ such that $\mu _{n_{j}}\Rightarrow \mu$ with the additional property that $r_{2}(n_{j})\rightarrow \infty$.

### Outline

For the convenience of the reader we briefly outline the contents of the paper. In Sect. 2 we give some explicit examples of attainable and non-attainable measures, and describe our motivation for studying the set of attainable measures. In Sect. 3 we give a brief background on Fourier coefficients of probability measures, and in Sect. 4 we recall some needed facts from number theory along with proving the more basic results above. Section 5 contains the proof of Theorem 1.3 (a complete classification of attainable measures in the region $|\hat{\mu }(4)|>1/3$), and Sect. 6 contains the proof of Theorem 1.6 (the complete classification of attainable measures in the region $|\hat{\mu }(4)| \le 1/3$), postponing some required results of technical nature to the appendix. Finally, in Sect. 7, we classify the set of square-free attainable measures.

## Examples of attainable and unattainable measures

### Some conventions

Let

$$\begin{aligned} \tilde{\delta }_{0} := \frac{1}{4}\sum _{k=0}^{3}\delta _{i^{k}} \end{aligned}$$

be the atomic probability measure supported at the 4 symmetric points $\pm 1$, $\pm i$ (“Cilleruelo measure”). Given an angle $\theta \in [0,\pi /4]$, let

9 $$\begin{aligned} \tilde{\delta }_{\theta } := \tilde{\delta }_{0} \bigstar ( \delta _{e^{i \theta }} + \delta _{e^{-i \theta }} )/2 = \frac{1}{8} \sum _{k=0}^{3} \left( \delta _{e^{i (\pi k/2+\theta )}} + \delta _{e^{i (\pi k/2-\theta )}} \right) ; \end{aligned}$$

recall that $\bigstar$ denotes convolution on $\mathcal {S}^{1}$. For $\theta =0,\pi /4$ the measure $\tilde{\delta }_{\theta }$ is supported at 4 points whereas for all other values of $\theta$ the support consists of 8 points. Given an integer $m \ge 1$ and $\theta \in [0,\pi /4]$, let

$$\begin{aligned} \tilde{\delta }_{\theta ,m} := \tilde{\delta }_{0} \bigstar \left( \frac{1}{m+1} \sum _{j=0}^{m}\delta _{e^{i \theta (m-2j)} } \right) . \end{aligned}$$

We note that $\tilde{\delta }_{\theta } = \tilde{\delta }_{\theta ,1}$, and that a measure $\mu$, a priori invariant under complex conjugation, is symmetric if and only if $\mu$ is invariant under convolution with $\tilde{\delta }_{0}$; in this case convolving with $\tilde{\delta }_{0}$ is a convenient way to ensure that a measure is symmetric.

### Some examples of attainable and unattainable measures

Given $\theta \in [0,\pi /4]$ let $\tau _{\theta }$ denote the symmetric probability measure with uniform distribution on the four arcs given by

$$\begin{aligned} \left\{ z : |z|=1, \arg (z) \in \cup _{k=0}^{4} [k\pi /2-\theta ,k\pi /2+\theta ]\right\} . \end{aligned}$$

Using some well known number theory given below (cf. Sect. 4) it is straightforward to show that $\tau _{\theta }$ is attainable for all $\theta \in [0,\pi /4]$. In particular, $d\mu _{\text {Haar}} = d\tau _{\pi /4}$, the Haar measure on $\mathcal {S}^{1}$ normalized to be a probability measure, is attainable. In fact, it is well known (see e.g.  [2]) that there exists a density one subsequence $\{n_{j}\}\subseteq S$, for which the corresponding lattice points $\Lambda _{n_{j}}$ become equidistributed on the circle; this gives another construction of $d\mu _{\text {Haar}}$ as an attainable measure.

It is also possible to construct other singular measures. In Sect. 4 we will outline a construction of attainable measures, uniformly supported on Cantor sets. Moreover, if *q* is a prime congruent to 3 modulo 4 it is well known that the solutions to $a^{2}+b^{2} = q^{2}$ are given by $(a,b) = (0,\pm q)$, or $(\pm q, 0)$, thus $\tilde{\delta }_{0}$ is attainable. A subtler fact, due to Cilleruelo, is that there exists sequences $\{n_{j}\}_{j\ge 1}$ for which $\Lambda _{n_{j}}$ has very singular angular distribution even though the number of points $r_{2}(n_{j})$ tends to infinity. Namely, it is possible to force all angles to be arbitrarily close to integer multiples of $\pi /2$, hence $\frac{1}{4}\sum _{k=0}^{3}\delta _{i^{k}}$ is an accumulation point of $d\mu _{n_{j}}$ as $n_{j} \rightarrow \infty$ in such a way that $r_{2}(n_{j}) \rightarrow \infty$.

We may also construct some explicit *unattainable* probability measures on $\mathcal {S}^{1}$ satisfying all the symmetries; in fact the following corollary of Theorem 1.6 constructs explicit unattainable measures, remarkably supported on 8 points only—the minimum possible for symmetric unattainable measures.

#### **Corollary 2.1**

(Corollary from Theorem 1.6) The probability measure

$$\begin{aligned} \eta _{a}:=a\tilde{\delta }_{0}+(1-a)\tilde{\delta }_{\pi /4} \end{aligned}$$

is attainable, if and only if $a= 0,\,\frac{1}{2},\,1$ or *a* is of the form

$$\begin{aligned} a=\frac{1}{2}\pm \frac{1}{2(2k+1)} \end{aligned}$$

for some $$k\ge 1$$.

## Fourier analysis of probability measures

### Some notation and de-symmetrization of probability measures

It is convenient to work with two models: either with the unit circle embedded in $\mathbb {C}$, or

$$\begin{aligned} \mathbb {T}^{1}:= {\mathbb R}/2\pi {\mathbb Z}. \end{aligned}$$

Rather than working with $\{ \mu _{n} \}$ and its weak partial limits, for notational convenience we work with their de-symmetrized variants, i.e.

10 $$\begin{aligned} d\nu _{n}(\theta ) = d\mu _{n}\left( \frac{\theta }{4}\right) , \end{aligned}$$

$\theta \in \mathbb {T}^{1}$. The measures $\nu _{n}$ are invariant under complex conjugation (recall that $\mathcal {S}^{1}\subseteq \mathbb {C}$); equivalently, for $\theta \in \mathbb {T}^{1}$,

$$\begin{aligned} d\nu _{n}(-\theta )=d\nu _{n}(\theta ). \end{aligned}$$

#### **Notation 3.1**

Let $\mathcal {P}$ be the set of all probability measures $\mu$ on $\mathcal {S}^{1}$ satisfying for $\theta \in \mathbb {T}^{1}$

11 $$\begin{aligned} d\mu (-\theta ) = d\mu (\theta ). \end{aligned}$$

Further, let $\mathcal {A}\subseteq \mathcal {P}$ be the set of all weak partial limits of $\{\nu _{n}\}$ i.e. all probability measures $\mu \in \mathcal {P}$ such that there exists a sequence $\{ n_{j}\}$ with

$$\begin{aligned} \nu _{n_{j}}\Rightarrow \mu . \end{aligned}$$

The set $\mathcal {A}$ defined above is the de-symmetrization of the collection of attainable measures via (10); by abuse of notation we will refer to the elements of $\mathcal {A}$ as attainable measures. One may restate Proposition 1.2 as stating that $\mathcal {A}$ is closed w.r.t. convolutions; thus $\mathcal {A}$ is an abelian monoid with identity $\delta _{0}\in \mathcal {A}$. The effect of the de-symmetrization (10) is that for all $m\in {\mathbb Z}$

$$\begin{aligned} \widehat{\nu _{n}}(m) = \widehat{\mu _{n}}(4m); \end{aligned}$$

since by the $\pi /2$-rotation invariance of $\mu _{n}$, $\widehat{\mu }(k) = 0$ unless *k* is divisible by 4, this transformation preserves all the information.

### Measure classification on the Fourier side

We would like to study the image of $\mathcal {A}$ under Fourier transform, or, rather, its projections into finite dimensional spaces. Since $\mathcal {A}\subseteq \mathcal {P}$ we first study the Fourier image of the latter; a proper inclusion of the image of $\mathcal {A}$ inside the image of $\mathcal {P}$ would automatically imply the existence of unattainable measures $\mu \in \mathcal {P}{\setminus }\mathcal {A}$.

For $\theta \in (0,\pi )$ let $\upsilon _{\theta }$ be the probability measure

12 $$\begin{aligned} \upsilon _{\theta } = \frac{1}{2}\left( \delta _{\theta }+\delta _{-\theta }\right) , \end{aligned}$$

and for the limiting values $\theta =0,\pi$ we denote $\upsilon _{0}=\delta _{0}$ and $\upsilon _{\pi } = \delta _{\pi }$. As for $\theta \in [0,\pi ]$, $\delta _{\theta }$ are the de-symmetrizations of $\tilde{\delta }_{\theta /4}$ in (9), attainable by Proposition 1.2 (see also Lemma 4.1 below), and it then follows that $\upsilon _{\theta }\in \mathcal {A}$. Clearly (see e.g.  [6, Chapter 1]) the set $\mathcal {P}$ is the convex hull of

$$\begin{aligned} \{\upsilon _{\theta }:\, \theta \in [0,\pi ]\}. \end{aligned}$$

Let $\mathcal {P}_{k}\subseteq {\mathbb R}^{k}$ be the image of $\mathcal {P}$ under the projection $\mathcal {F}_{k} : \mathcal {P}\rightarrow {\mathbb R}^{k}$ given by

$$\begin{aligned} \mathcal {F}_{k}(\mu ) :=(\widehat{\mu }(1),\ldots , \widehat{\mu }(k)), \end{aligned}$$

i.e. $\mathcal {P}_{k}=\mathcal {F}_{k}(\mathcal {P})$ are the first *k* Fourier coefficients of the measure $\mu$ as $\mu$ varies in $\mathcal {P}$. Recalling the invariance (11) for $\mu \in \mathcal {P}$ we may write

$$\begin{aligned} \mathcal {F}_{k}\mu = (\widehat{\mu }(1),\ldots , \widehat{\mu }(k)) = \int \limits _{0}^{2\pi }\gamma _{k}(\theta )d\mu (\theta ), \end{aligned}$$

where $\gamma _{k}$ is the curve

$$\begin{aligned} \gamma _{k}(\theta ) = (\cos (\theta ),\cos (2\theta ),\ldots ,\cos (k\theta )) \end{aligned}$$

for $\theta \in [0,2\pi ]$. Thus $\mathcal {P}_{k}=\mathcal {F}_{k}(\mathcal {P})$ could be regarded as a convex combination of points lying on $\gamma _{k}$ (corresponding to $\upsilon _{\theta }$); it would be then reasonable to expect $\mathcal {P}_{k}$ to be equal to the convex hull of $\gamma _{k}$.

This intuition was made rigorous in a more general scenario by F. Riesz  [7] in a classical theorem on the generalized moments problem (cf.  [6], Chapter 1, Theorem 3.5 on p. 16). The sets $\mathcal {P}_{k}$ are the convex hulls of the curves $\gamma _{k}$ in ${\mathbb R}^{k}$ indeed. Interestingly, since $\cos (m\theta )$ is a polynomial in $\cos (\theta )$, the curve $\gamma _{k}$ is algebraic. As a concrete example, for $k=2$ the image $\mathcal {P}_{2}$ of $\mathcal {P}$ under

$$\begin{aligned} \mathcal {F}_{2}:\mu \mapsto (\widehat{\mu }(1),\widehat{\mu }(2)) \end{aligned}$$

is the convex hull of the parabola $y=2x^{2}-1$, $x\in [-1,1]$, i.e. the set

13 $$\begin{aligned} \mathcal {P}_{2} = \{(x,y): x\in [-1,1],\,2x^{2}-1\le y \le 1 \} , \end{aligned}$$

as shown in Fig. 1, to the left.

Analogously to the above, define

$$\begin{aligned} \mathcal {A}_{k}=\mathcal {F}_{k}(\mathcal {A})\subseteq \mathcal {P}_{k}, \end{aligned}$$

(cf. (5), and bear in mind the de-symmetrization (10)). Since, by the definition, $\mathcal {A}$ is closed in $\mathcal {P}$ (i.e. the weak limit set of $\mathcal {A}$ satisfies $\mathcal {A}'\subseteq \mathcal {A}$), if follows that for every $k\ge 2$, $\mathcal {A}_{k}$ is closed in $\mathcal {P}_{k}$ in the usual sense. The shell $y=2x^{2}-1$ of the convex hull $\mathcal {P}_{2}$ is (uniquely) attained by the family $\{\upsilon _{\theta }\ :\theta \in [0,\pi ]\}$ of measures as in (12) with the Fourier coefficients

14 $$\begin{aligned} (\widehat{\upsilon _{\theta }}(1),\widehat{\upsilon _{\theta }}(2))=(\cos (\theta ),\cos (2\theta )). \end{aligned}$$

Finally, it is worth mentioning that the set $\mathcal {A}$ is *not convex*, as $\mathcal {A}_{2}$ contains the parabola

$$\begin{aligned} \{(x,2x^{2}-1):\, x\in [-1,1]\} \subseteq \mathcal {A}_{2}, \end{aligned}$$

whose points correspond to the measures (12), though not its convex hull. (In other words, had $\mathcal {A}$ been convex, that would force all symmetric measures to be attainable.)

## Proofs of the basic results

### Number theoretic background

We start by giving a brief summary on the structure of $\Lambda _{n}$ (equivalently, $\mu _{n}$ or their de-symmetrized by (10) versions $\nu _{n}$) given the prime decomposition of *n*. These results follow from the (unique) prime factorization of Gaussian integers, see e.g.  [1]. First, for every “split” prime

$$\begin{aligned} p\equiv 1 \mod 4, \end{aligned}$$

there exists an angle $\theta _{p}\in [0,\pi ]$, such that the measure $\nu _{p}$ arising from *p* is given by

$$\begin{aligned} \nu _{p} = \upsilon _{\theta _{p}}=(\delta _{\theta _p} + \delta _{-\theta _p} )/2. \end{aligned}$$

More generally, if a split prime *p* occurs to a power $p^{e}$, we find that the resulting measure is given by

$$\begin{aligned} \nu _{p^e} = \upsilon _{\theta _{p},e}, \end{aligned}$$

where

15 $$\begin{aligned} \upsilon _{\theta ;M}= \frac{1}{M+1}\sum \limits _{k=0}^{M}\delta _{(M-2k)\theta }, \end{aligned}$$

and hence, in particular,

$$\begin{aligned} r_{2}(p^{e}) = 4(e+1) \end{aligned}$$

(recall the de-symmetrization (10)). Both the $\{\nu _{n}\}$ and $\frac{1}{4}r_{2}(n)$ are multiplicative in the sense that for $n_{1},n_{2}$ co-prime numbers $(n_{1},n_{2})=1$,

16 $$\begin{aligned} \nu _{n_{1}\cdot n_{2}} = \nu _{n_{1}}\bigstar \nu _{n_{2}}, \end{aligned}$$

and

$$\begin{aligned} r_{2}(n_{1})r_{2}(n_{2})=4r_{2}(n_{1} n_{2}). \end{aligned}$$

In particular, $r_{2}(n)=0$ unless *n* is of the form

$$\begin{aligned} n=2^{a}p_{1}^{e_{1}}\cdot \ldots \cdot p_{k}^{e_{k}}q_{1}^{2r_{1}}\cdot \ldots \cdot q_{l}^{2r_{l}}, \end{aligned}$$

for $p_{i}\equiv 1\mod 4$, $q_{j}\equiv 3\mod 4$ primes (in particular, all the exponents of primes $\equiv 3 \mod 4$ are even); in this case

$$\begin{aligned} \nu _{n} = \bigstar _{i=1}^{k} \nu _{p_{i}^{e_{i}}}, \end{aligned}$$

and

$$\begin{aligned} r_{2}(n) = 4\prod \limits _{i=1}^{k}(e_{i}+1). \end{aligned}$$

By Hecke’s celebrated result  [3, 4] the angles $\theta _{p}$ are equidistributed in $[0,\pi /4]$: for every $0 \le \alpha < \beta \le \pi$,

$$\begin{aligned} \#\{ p \le X, \, p\equiv 1(4): \, \theta _{p}\in [\alpha ,\beta ] \} \sim \frac{(\beta -\alpha )}{\pi /4} \cdot \frac{X}{2\log {X}} \end{aligned}$$

In particular, the following lemma is an immediate consequence.

#### **Lemma 4.1**

For every $\theta \in [0,\pi ]$ and $\epsilon >0$ there exist a split prime *p* with

$$\begin{aligned} |\theta _{p}-\theta |<\epsilon . \end{aligned}$$

### Proof of Proposition 1.2

#### *Proof*

We will prove the equivalent de-symmetrized version of the statement, i.e. that if $\gamma _{1},\gamma _{2}\in \mathcal {A}$ then

$$\begin{aligned} \gamma _{1}\bigstar \gamma _{2}\in \mathcal {A}. \end{aligned}$$

Let $\{m_{k}\},\{n_{k} \}\subseteq S$ be two sequences so that $\nu _{m_{k}}\Rightarrow \gamma _{1}$, $\nu _{n_{k}}\Rightarrow \gamma _{2}$. We would like to invoke the multiplicativity (16) of $\{ \nu _{n} \}$; we cannot apply it directly, as $n_{k}$ and $m_{k}$ may fail to be co-prime. To this end rather than using $\nu _{m_{k}}$ we are going to substitute^1^ it with $\nu _{m'_{k}}$ chosen to approximate $\nu _{m_{k}}$, so that $m'_{k}$ is co-prime to $n_{k}$, via Lemma 4.1. In the remaining part of the proof we shall argue that

17 $$\begin{aligned} \nu _{m'_{k}\cdot n_{k}} = \nu _{m'_{k}} \bigstar \nu _{n_{k}} \Rightarrow \gamma _{1}\bigstar \gamma _{2}, \end{aligned}$$

provided we care to choose $m'_{k}$ so that $\nu _{m'_{k}}$ approximates $\nu _{m_{k}}$ sufficiently well.

To this end it is more convenient to work with the space of Fourier coefficients; the weak convergence of probability measures corresponds to point-wise convergence of the Fourier coefficients. By Lemma 4.1 we may replace $m_{k}$ with $m'_{k}$ co-prime to $n_{k}$ that satisfies for every $j \le k$

$$\begin{aligned} \left| \widehat{\nu _{m_{k}}}(j) - \widehat{\nu _{m'_{k}}}(j)\right| < \frac{1}{k}. \end{aligned}$$

It then readily follows that $\nu _{m'_{k}} \Rightarrow \gamma _{1}$, and hence we establish (17), which in turn implies that $\gamma _{1}~\bigstar ~ \gamma _{2} \in \mathcal {A}$, finally yielding the closedness of $\mathcal {A}$ w.r.t. convolutions.

As for the second assertion, if $\mu \in \mathcal {A}$, then $\mu$ is a weak limit of a sequence $\nu _{n_{j}}$ for some $\{n_{j}\}\subseteq S$. Factoring $n_{j} = p_{j;1}^{e_{j;1}} \cdot \ldots \cdot p_{j;r}^{e_{j;r}}$ we have

$$\begin{aligned} \nu _{n_{j}} = \nu _{p_{j;1}^{e_{j;1}}}\bigstar \ldots \bigstar \nu _{p_{j;r}^{e_{j;r}}} \end{aligned}$$

and thus $\mu$ indeed lies in the closure of finite convolutions of prime power attainable measures of the form $\nu _{p^{e}}$. $\square$

#### *Remark 4.2*

The proof of Theorem 1.4 is similar—replacing prime power attainable measures with prime attainable measures in the above argument yields the corresponding result for square-free attainable measures.

### Cantor sets are attainable 

By Proposition 1.2, $\mathcal {A}$ is closed under convolution, it contains  [5] uniform measures supported on symmetric intervals $[-\theta ,\theta ]$, as well as symmetric sums $(\delta _\theta + \delta _{-\theta })/2$ for all $\theta >0$. Thus, by using an “additive” construction of Cantor sets, we easily see that uniform measures supported on Cantor sets are attainable.

Namely, given $\theta >0$, let $C_{n,\theta }$ be the *n*-th level Cantor set obtained by starting with the interval $[-\theta , \theta ]$ and deleting the middle third part of the interval: $C_{0,\theta }$ consists of one closed interval $[-\theta , \theta ]$, and $C_{n+1,\theta } \subset C_{n,\theta }$ is the union of the $2^{n+1}$ intervals obtained by removing the middle third in each of the $2^{n}$ intervals that $C_{n,\theta }$ consists of. Now,

18 $$\begin{aligned} C_{n+1,\theta } = (C_{n,\theta /3} - 2\theta /3) \sqcup (C_{n,\theta /3} + 2\theta /3), \end{aligned}$$

where $\sqcup$ denotes disjoint union, and $C_{n+1,\theta /3} + \alpha$ denotes the translation of the set $C_{n+1,\theta /3}$ by $\alpha$.

Since $C_{0,\theta }$ is a symmetric interval, the measure corresponding to its characteristic function is attainable, as mentioned above. Further, since convolving $(\delta _\theta + \delta _{-\theta })/2$ with a uniform measure having support on some set *D* yields a measure with support on $(D + \theta ) \cup (D-\theta )$, uniform measures supported on $C_{n,\theta }$ are attainable by induction, via (18). Letting $n \rightarrow \infty$ we find that measures with uniform support on Cantor sets are attainable.

### Proof of Proposition 1.7

#### *Proof*

We are going to make use of a (de-symmetrized) Cilleruelo sequence $n_{j}$, i.e. $\nu _{n_{j}}\Rightarrow \delta _{0}$ and $r_{2}(n_{j})\rightarrow \infty$. Let $\mu \in \mathcal {A}$ be an attainable measure and assume that $\nu _{m_{j}}\Rightarrow \mu$. Using the same idea as in the course of proof of Proposition 1.2 above we may assume with no loss of generality that $(n_{j},m_{j})=1$ are co-prime (recall that $\{ n_{j}\}$ is a Cilleruelo sequence of our choice). Then

$$\begin{aligned} \nu _{m_{j}\cdot n_{j}} = \nu _{m_{j}}\bigstar \nu _{n_{j}} \Rightarrow \mu \bigstar \delta _{0} = \mu , \end{aligned}$$

and

$$\begin{aligned} r_{2}(m_{j}\cdot n_{j})/4 = r_{2}(m_{j})\cdot r_{2}(n_{j}) \rightarrow \infty , \end{aligned}$$

so that the sequence $\{n_{j}\cdot m_{j}\}$ is as required. $\square$

## Proof of Theorem 1.3: measure classification for $x>\frac{1}{3}$

### Some conventions related to Fourier analysis

We adapt the following conventions. The *k*-th Fourier coefficient of a measure $\mu \in \mathcal {P}$ is given by

$$\begin{aligned} \widehat{\mu }(k)= \int \limits _{\mathbb {T}^{1}}\cos (k\theta )d\mu (\theta ); \end{aligned}$$

clearly $|\widehat{\mu }(k)|\le 1$. The convolution of two probability measures $\mu ,\mu '\in \mathcal {P}$ is the probability measure $\mu \bigstar \mu '$ defined as

$$\begin{aligned} d(\mu \bigstar \mu ') (\theta ) = \int \limits _{\mathbb {T}^{1}}d\mu (\theta ')d\mu '(\theta -\theta '). \end{aligned}$$

With the above conventions we have

$$\begin{aligned} \widehat{\mu \bigstar \mu '}(k)=\widehat{\mu }(k)\cdot \widehat{\mu '}(k). \end{aligned}$$

It is easy to compute the Fourier coefficients of $\upsilon _{\theta ;M}$ as in (15) to be

$$\begin{aligned} \widehat{\upsilon }_{\theta ;M}(k) = \frac{1}{M+1}\sum \limits _{j=0}^{M}\cos ((M-2j)k\theta )= G_{M+1}(k\theta ), \end{aligned}$$

where

19 $$\begin{aligned} G_{A}(\theta ) := \frac{\sin (A\theta )}{A\sin \theta }; \end{aligned}$$

for $M=1$, $G_{2}(\theta )=\cos (\theta )$ is consistent with (14).

By the definition of $\mathcal {A}$ and $\mathcal {A}_{k}=\mathcal {F}_{k}(\mathcal {A})$ and in light of Lemma 4.1, we can describe $\mathcal {A}_{k}$ geometrically as the smallest multiplicative set, closed in $\mathcal {P}_{k}$, containing all the curves^2^

$$\begin{aligned} \left\{ \gamma _{k;A}(\theta ):=(G_{A}(\theta ),\,\ldots ,G_{A}(k\theta )):\,\theta \in [0,\pi ] \right\} _{A\ge 2}, \end{aligned}$$

i.e. $\mathcal {A}_{k}$ is the closed multiplicative subset of $\mathcal {P}_{k}$ generated by the above curves. Similarly, the set corresponding to the square-free attainable measures $\mathcal {A}^{0}_{k}$ is the smallest closed multiplicative set containing the single curve

$$\begin{aligned} \gamma _{k;2}(\theta )=(\cos (\theta ),\,\ldots ,\cos (k\theta )), \end{aligned}$$

$$\theta \in [0,\pi ]$$.

From this point on we will fix $k=2$ and suppress the *k*-dependence in the various notation, e.g. $\gamma _{A}$ will stand for $\gamma _{2;A}$. The curves

20 $$\begin{aligned} \gamma _{A}(\theta ):=(G_{A}(\theta ),G_{A}(2\theta )) \end{aligned}$$

for $2\le A\le 20$ are displayed in Fig. 4, separately for odd and even $M=A-1$.

### Proof of Theorem 1.3

The two statements of Theorem 1.3 are claimed in Propositions 5.1 and 5.2, and proved in Sects. 5.3 and 5.6 respectively. Note that Proposition 5.2 yields attainable measures with the relevant Fourier coefficients regardless whether $x>\frac{1}{3}$ or $x\le \frac{1}{3}$.

#### **Proposition 5.1**

Points (*x*, *y*) with $x>\frac{1}{3}$ corresponding to attainable measures lie under the max curve, i.e. if $(x,y)\in \mathcal {A}_{2}$ then

21 $$\begin{aligned} y \le \mathcal {M}(x), \end{aligned}$$

where $\mathcal {M}(x)$ is given by (3).

#### **Proposition 5.2**

Given *x*, *y* such that $|x| \le 1$ and

$$\begin{aligned} 2x^{2}-1 \le y \le \mathcal {M}(x) , \end{aligned}$$

there exists an attainable measure $\mu$ such that $( \hat{\mu }(4), \hat{\mu }(8)) = (x,y)$.

### Proof of Proposition 5.1: attainable measures lie under the max curve for $x>1/3$

In what follows, by componentwise product we will mean

22 $$\begin{aligned} (x_{1},y_{1})\cdot (x_{2},y_{2}) = (x_{1}\cdot x_{2}, y_{1} \cdot y_{2}). \end{aligned}$$

#### **Definition 5.3**

(*Totally positive and mixed sign points*) Let $\mathcal {A}_{2}^{+}\subseteq \mathcal {A}_{2}$ be the set of **totally positive** attainable points admitting a representation as finite componentwise products

23 $$\begin{aligned} (x,y) = \prod \limits _{i=1}^{K}(x_{i},y_{i}) \end{aligned}$$

of points $(x_{i},y_{i})=\gamma _{2;A_{i}}(\theta _{i})$ for some $A_{i}\ge 2$, $\theta _{i}\in [0,\pi ]$, so that for all $i \le K$ we have $y_{i}>0$. Similarly, $\mathcal {A}_{2}^{-}\subseteq \mathcal {A}_{2}$ is the set of **mixed sign** attainable points admitting representation (23) with at least one $y_{i}<0$.

Note that a point in $\mathcal {A}_{2}$ may be both totally positive and of mixed sign, i.e. $\mathcal {A}_{2}^{+}$ may intersect $\mathcal {A}_{2}^{-}$. Furthermore, a priori it may be in neither of these. However, by the definition of $\mathcal {A}_{2}$, it is the closure of the union of the sets defined:

24 $$\begin{aligned} \overline{\mathcal {A}_{2}^{+}\cup \mathcal {A}_{2}^{-}} = \mathcal {A}_{2}. \end{aligned}$$

Therefore to prove the inequality (21) on $\mathcal {A}_{2}$ it is sufficient to prove the same for points in $\mathcal {A}_{2}^{+}$ and $\mathcal {A}_{2}^{-}$ separately. These are established in Lemma 5.4 and Proposition 5.5, proved in Sects. 5.4 and 5.5 respectively.

#### **Lemma 5.4**

If $(x,y)\in \mathcal {A}_{2}^{-}$ is a mixed sign attainable point then

$$\begin{aligned} y \le (2|x|-1)^{2}. \end{aligned}$$

#### **Proposition 5.5**

Let $(x,y)=\gamma _{A}(\theta )$ for some $A\ge 2$ and $\theta \in [0,\pi ]$ such that $x>\frac{1}{3}$. Then $y \le x^{4}$.

We are now in a position to prove Proposition 5.1.

#### *Proof of Proposition 5.1 assuming Lemma 5.4 and Proposition 5.5*

If the point $(x,y) \in \mathcal {A}_{2}^{-}$ is of mixed sign, Lemma 5.4 applies and hence $y \le (2|x|-1)^{2}$. Otherwise, if the point is totally positive,

$$\begin{aligned} (x,y) = \left( \prod _{i} x_{i}, \prod _{i}y_{i}\right) \end{aligned}$$

where $(x_{i},y_{i})$ are prime power attainable, and $y_{i} \ge 0$ for all *i*.

Now, $|x_{i}| \le 1$ for all *i* since $x_{i}$ is a Fourier coefficient of a probability measure, so if $|x|>1/3$ we must have $|x_{i}| > 1/3$ for all *i*. By Proposition 5.5, $y_{i} \le x_{i}^{4}$ for all *i*, and thus $y \le x^{4}$. Thus it follows that the statement (21) of Proposition 5.1 holds on $\mathcal {A}_{2}^{+}\cup \mathcal {A}_{2}^{-}$ and thus on its closure, $\mathcal {A}_{2}$ (cf. (24)). $\square$

### Proof of Lemma 5.4: the mixed sign points $\mathcal {A}_{2}^{-}$ lie under the max curve

To pursue the proof of Lemma 5.4 we will need some further notation.

#### **Notation 5.6**

Let $B_{1}\subseteq [-1,1] \times [-1,1]$ be the set

$$\begin{aligned} B_{1} = \{(x,y):\, x \in [-1/2,1/2],\, 0 \le y \le (2|x|-1)^{2} \}, \end{aligned}$$

and $B\subseteq [-1,1] \times [-1,1]$ be the domain

$$\begin{aligned} B_{2} = \{(x,y):\, x\in [-1/\sqrt{2},1/\sqrt{2}],\, 2x^{2}-1 \le y \le 0\}. \end{aligned}$$

Recall the Definition 5.3 of totally positive attainable points $\mathcal {A}_{2}^{+}$, and componentwise product of points (22). It is obvious that the points of either $B_{1}$ and $B_{2}$ are all lying under the max curve, i.e. if

$$\begin{aligned} (x,y)\in B_{1}\cup B_{2}, \end{aligned}$$

then

$$\begin{aligned} y \le \mathcal {M}(x). \end{aligned}$$

Therefore the following lemma implies Lemma 5.4.

#### **Lemma 5.7**

If $(x,y)\in \mathcal {A}_{2}^{-}$ is a mixed sign attainable point then

$$\begin{aligned} (x,y) \in B_{1}\cup B_{2}. \end{aligned}$$

To prove Lemma 5.7 we establish the following two auxiliary lemmas whose proof is postponed until immediately after the proof of Lemma 5.7.

#### **Lemma 5.8**

If $(x,y) = (\hat{\mu }(1), \hat{\mu }(2))$ for $\mu$ some probability measure on ${\mathcal {S}^{1}}$ and $y \le 0$, then $(x,y) \in B_{2}$.

#### **Lemma 5.9**

If $$p_{1}, p_{2} \in B_{2}$$, then $$p_{1} \cdot p_{2} \in B_{1}$$.

#### *Proof of Lemma 5.7 assuming the auxiliary lemmas*

Let

$$\begin{aligned} (x,y)\in \mathcal {A}_{2}^{-} \end{aligned}$$

be given. First, if $(x,y)\in \mathcal {A}_{2}^{-}$ with $y\le 0$, then $(x,y)\in B_{2}$ by Lemma 5.8; hence we may assume $y>0$. Let $(x_{i},y_{i})$ be as in (23), which according to the Definition 5.3 have mixed signs. Since $y \ge 0$ we can in fact find $i \ne j$ for which $y_{i},y_{j}< 0$, and without loss of generality we may assume that $(i,j)=(1,2)$. Letting

$$\begin{aligned} (\tilde{x},\tilde{y}) = \left( \prod _{k \ne 1,2} x_{k}, \prod _{k \ne 1,2} y_{k}\right) \end{aligned}$$

we find that

$$\begin{aligned} (x,y) = (x_{1},y_{1})\cdot (x_{2},y_{2}) \cdot (\tilde{x},\tilde{y}), \end{aligned}$$

where $$\tilde{y} \in [0,1]$$ and $$\tilde{x} \in [-1,1]$$.

We further note that both $(x_{1},y_{1})$ and $(x_{2},y_{2})$ lie in $B_{2}$. Thus by Lemma 5.9,

$$\begin{aligned} (x_{1},y_{1})\cdot (x_{2},y_{2}) \in B_{1}. \end{aligned}$$

Since $|\tilde{x}|,\tilde{y} \le 1,$ the result follows on noting that $B_{1}$ is mapped into itself by any map of the form

$$\begin{aligned} (x,y) \rightarrow (\alpha x, \beta y), \end{aligned}$$

provided that

$$\begin{aligned} 0 \le |\alpha |, \beta \le 1. \end{aligned}$$

$$\square $$

#### Proofs of the auxiliary Lemmas 5.8 and 5.9

##### *Proof of Lemma 5.8*

The assumptions are equivalent to $(x,y)\in \mathcal {P}_{2}$ with $y\le 0$. The statement follows immediately upon using the explicit description (13) of $\mathcal {P}_{2}$:

$$\begin{aligned} \mathcal {P}_{2}\cap \{ y\le 0\} = B_{2}. \end{aligned}$$

$$\square $$

##### *Proof of Lemma 5.9*

The case of either point having zero *y*-coordinate is trivial, so we may assume that both $p_{1},p_{2}$ have negative *y*-coordinates, and it suffices to prove the statement for points $p_{1},p_{2}$ having minimal *y*-coordinates, i.e.,

$$\begin{aligned} p_{1} = (a, 2a^{2}-1), \quad p_{2} = (b, 2b^{2}-1), \end{aligned}$$

and we may further assume $ab\ne 0$ as otherwise the statement is trivial.

By symmetry it suffices to consider the case $a,b \in (0,1/\sqrt{2})$. Thus, if we fix $c \in (0,1/2)$ it suffices to determine the maximum of

$$\begin{aligned} (2a^{2}-1)(2b^{2}-1) \end{aligned}$$

subject to the constraint $ab = c$. Taking logs we find that the constraint is given by

$$\begin{aligned} \log a + \log b = \log c \end{aligned}$$

and we wish to maximize

$$\begin{aligned} \log ( 1-2a^{2}) + \log (1-2b^{2}). \end{aligned}$$

Using Lagrange multipliers we find that all internal maxima satisfies

$$\begin{aligned} (1/a,1/b) = \lambda \left( \frac{4a}{1-2a^{2}}, \frac{4b}{1-2b^{2}}\right) \end{aligned}$$

for some $\lambda \in {\mathbb R}$. If $c = ab \ne 0$ we find that

$$\begin{aligned} (1,1) = \lambda \left( \frac{4a^{2}}{1-2a^{2}}, \frac{4b^{2}}{1-2b^{2}}\right) \end{aligned}$$

and thus $\frac{4a^{2}}{1-2a^{2}} = \frac{4b^{2}}{1-2b^{2}}$ which implies that $a^{2}=b^{2}$, and hence, recalling that we assumed $a,b\ge 0$, it yields $a = b$. In particular, any internal maximum gives a point $(a^{2}, (2a^{2}-1)^{2}) = (c,(2|c|-1)^{2}$), which lies on the boundary of $B_{1}$. As mentioned earlier, for points on the boundary, the inequality holds trivially. $\square$

### Proof of Proposition 5.5: totally positive points $\mathcal {A}_{2}^{+}$ corresponding to prime powers

#### **Lemma 5.10**

The function $\frac{\sin t }{t}$ is decreasing and is $\ge 0$ on $[0,\pi ]$.

#### *Proof*

Taking derivatives, this amounts to the fact that $\tan t > t$ on $(0,\pi /2)$. $\square$

#### **Lemma 5.11**

If $A \ge 4$ and $|G_{A}(t)| \ge 1/3$ for $t \in [0,\pi /2]$, then $t \le \frac{\pi }{A}$. For $A=3$, we have the further possibility that $t = 3 \pi /(2A) = \pi /2$.

#### *Proof*

The inequality $\sin t \ge 2t/\pi$, valid for $t \in [0,\pi /2]$, and strict except at the end points, gives that

$$\begin{aligned} |G_{A}(t) | = \left| \frac{\sin (At)}{A\sin {t}} \right| \le \frac{1}{A \sin t} \le \frac{1}{A \cdot \frac{2}{\pi } t} \end{aligned}$$

and hence $|G_{A}(t)| < 1/3$ for $t > 3 \pi /(2A)$, for any $A>0$. It thus suffices to consider $t \in [0, 3 \pi /(2A)]$.

Consider first the case $A=3$. We begin by showing that $G_{3}(t)$ is decreasing on $[0,\pi /2]$. Taking derivatives, this amounts to the fact that

$$\begin{aligned} 3 \tan t \ne \tan 3t \end{aligned}$$

on $(0,\pi /2)$ (note that the derivative is negative for $t=\pi /6$). Now, since $G_{3}(\pi /3) = 0$ and $G_{3}(\pi /2) = -1/3$ and $G_{3}$ is decreasing, we find that the only possibility for $|G_{3}(t)| = 1/3$ and $t \in [\pi /3,\pi /2]$ is $t = \pi /2$. Thus, any other solution must lie in $[0,\pi /3] = [0,\pi /A]$.

For $A \ge 4$, note that

25 $$\begin{aligned} \left| \frac{\sin At}{A \sin t} \right| = \left| \frac{\sin (At)/(At)}{\sin (t)/t} \right| < \left| \frac{\sin (At)/(At)}{\sin (At/3)/(At/3)} \right| \end{aligned}$$

(for $t \le 3\pi /(2A)$ we have $At/3 \le \pi /2$, hence

$$\begin{aligned} |\sin (At/3)/(At/3) | \le |\sin (t)/t|, \end{aligned}$$

since $(\sin x)/x$ is decreasing on the interval $[0,\pi ]$ by Lemma 5.10.)

Taking $s = At/3$, the RHS of (25) becomes

$$\begin{aligned} \frac{(\sin 3s)/3s}{(\sin s) /s} = \frac{\sin 3s}{3 \sin s} \end{aligned}$$

and $t \le 3\pi /(2A)$ implies that $s \le \pi /2$. For this range of *s*, by the first part of the lemma, we find that $\left| \frac{\sin 3s}{3 \sin s}\right| \ge 1/3$ implies that either $s=\pi /2$ or $s \le \pi /3$, which in turn implies that $t =3\pi /(2A)$ or $t \le \pi /A$. Noting that the first possibility is ruled out by the strict inequality in (25), the proof is concluded. $\square$

We proceed to characterize points lying on curves $\{ (x,y)=\gamma _{A}(t)\}_{A\ge 2}$, for which $x > 1/3$ and $y \ge 0$, showing that any such point satisfies $y \le x^{4}$. We begin with the following key Lemma.

#### **Lemma 5.12**

For $$t \in (0,\pi /2]$$, define

26 $$\begin{aligned} h(t) := \frac{t^{3}\cos t}{\sin ^{3}t} \end{aligned}$$

and extend *h* to $[0,\pi /2]$ by continuity. Then *h*(*t*) is decreasing on $[0, \pi /2]$.

#### *Proof*

We have

$$\begin{aligned} h'(t) = \frac{t^{2}\sin ^{2}(t) \left( \sin (t) \cos (t)-t\sin ^{2}(t)-3t\cos ^{2}(t)\right) }{\sin ^{6}t}, \end{aligned}$$

and it is enough to show that

27 $$\begin{aligned} \sin (t) \cos (t)-t\sin ^{2}(t)-3t\cos ^{2}(t) < 0 \end{aligned}$$

for $t\in (0,\pi /2)$. Since for $t=0$ the expression on the left hand side of (27) vanishes it is sufficient to show that its derivative is strictly negative on $\left( 0,\frac{\pi }{2}\right)$. We find that

$$\begin{aligned}&\left( \sin (t) \cos (t)-t\sin ^{2}(t)-3t\cos ^{2}(t) \right) ' \\&\quad = 4\sin (t)(t\cos (t)-\sin (t)) =4\sin (t) \cos (t)(t-\tan {t}) < 0 \end{aligned}$$

since $$\tan (t)>t$$ on $$\left( 0,\frac{\pi }{2}\right) $$. $$\square $$

#### *Proof of Proposition 5.5*

If $A=2$, the points lying on the curve $\gamma _{2}$ are of the form

$$\begin{aligned} (x,y)=\gamma _{2}(t)= (t, 2t^{2}-1), \end{aligned}$$

and it is straightforward to check that $2t^{2}-1 \le t^{4}$. For $A \ge 3$, since we assume that $x > 1/3$ and

$$\begin{aligned} (x,y) = (G_{A}(t), G_{A}(2t) ), \end{aligned}$$

Lemma 5.11 implies that $t \le \pi /A$. In fact, $t \le \pi /(2A)$, as we assume that $y \ge 0$. Hence it is sufficient to show that

$$\begin{aligned} \frac{\sin 2At}{A \sin 2t} \le \left( \frac{\sin At}{A \sin t}\right) ^{4} \end{aligned}$$

holds for $$t \in [0, \pi /(2A)]$$.

This in turn is equivalent (note that all individual trigonometric terms are non-negative since $t \in [0, \pi /(2A)]$) to

$$\begin{aligned} A^{3} \cos At \sin ^{3} t \le \sin ^{3} At \cos t \end{aligned}$$

which is equivalent to

$$\begin{aligned} \frac{(At)^{3} \cos At}{\sin ^{3} At} \le \frac{t^{3 }\cos t}{\sin ^{3} t}. \end{aligned}$$

Setting

$$\begin{aligned} s = At\in [0,\pi /2], \end{aligned}$$

we find that this is equivalent to

$$\begin{aligned} \frac{s^{3} \cos s}{\sin ^{3} s} \le \frac{(s/A)^{3 }\cos s/A}{\sin ^{3} s/A}, \end{aligned}$$

or, equivalently on recalling (26), that

$$\begin{aligned} h(s) \le h(s/A). \end{aligned}$$

which, as $A>1$, follows from Lemma 5.12. $\square$

### Proof of Proposition 5.2: all points under the max curve are attainable

#### **Lemma 5.13**

The curve $\{ (x,x^4): x\in [0,1]\}$ is square-free attainable, i.e. all the points on this curve correspond to at least one attainable measure.

#### *Proof of Proposition 5.2 assuming Lemma 5.13*

By the definition of the max curve (3) it is sufficient to prove that if $(x_{0},y_{0})$ is lying under one of the curves $y=x^{4}$ and $y=(2|x|-1)^{2}$ then $(x_{0},y_{0})\in \mathcal {A}_{2}$ is attainable; with no loss of generality we may assume that $x_{0}\ge 0$. Now we know that the parabola $\{ (t,2t^{2}-1)\}_{t\in [0,1]}$ is attainable, and from Lemma 5.13 so is the curve $\{(x,x^{4})\}_{x\in [0,1]}$.

It then follows by multiplicativity of $\mathcal {A}_{2}$ that all the points of the form

$$\begin{aligned} (x_{0},y_{0}) = (x,x^{4}) \cdot (t,2t^{2}-1) \end{aligned}$$

are attainable (recalling the notation (22) for componentwise multiplication). On the other hand it is clear that the union of the family of the parabolas

$$\begin{aligned} \{(xt,x^{4}(2t^{2}-1)) : t \in [0,1] \}, \end{aligned}$$

as *x* ranges over [0, 1], is exactly the set

$$\begin{aligned} \{(x,y):\, x\in [0,1], \, 2x^{2}-1 \le y\le x^{4} \}. \end{aligned}$$

Concerning points under the other curve $y=(2x-1)^2$ we may employ the multiplicativity of $\mathcal {A}_{2}$ again to yield that the curve

$$\begin{aligned} \{(x^{2},(2x^{2}-1)^{2})\}_{x\in [0,1]} \end{aligned}$$

is attainable; this curve in turn can be re-parameterized as $\{ (t,(2t-1)^{2})\}_{t\in [0,1]}$. A similar argument to the above shows that function

$$\begin{aligned} (x,t)\mapsto (x,(2x-1)^{2})\cdot (t,2t^{2}-1) \end{aligned}$$

maps $[0,1]^{2}$ onto the domain

$$\begin{aligned} \{(x,y):\, x\in [0,1],\, 2x^{2}-1 \le y\le (2x-1)^{2} \}, \end{aligned}$$

i.e. as the parameter *x* varies along [0, 1] the parabolas

$$\begin{aligned} \{(xt,(2x-1)^{2}\cdot (2t^{2}-1))\} \end{aligned}$$

tessellate the domain under the curve $y=(2x-1)^{2}$, $x\in [0,1]$. Hence all the points under the latter curve are attainable, as claimed. $\square$

#### *Proof of Lemma 5.13*

We start with the case $x \ge 0$. We know that the curve $\{(x, 2x^2-1)\}_{x\in [-1,1]}$ is attainable as a re-parametrization of $(\cos \theta , \cos 2 \theta )$ (i.e. all the points on that curve correspond to attainable measures), hence for $n\ge 1$ the curve $\{(x^n, (2x^2-1)^n)\}$ is attainable by the multiplicativity (cf. Proposition 1.2). Fix $\alpha >0$, and take $x = x_n = e^{-\alpha / n}$. Thus

$$\begin{aligned} ( e^{-\alpha }, (2 e^{-2 \alpha /n} - 1)^n ) \end{aligned}$$

is attainable for every $\alpha > 0$ and $n \ge 1$.

Upon using Taylor series, we find that, as $n \rightarrow \infty$,

$$\begin{aligned} (2 e^{-2 \alpha /n} - 1)^n= & {} \left( 2 \left( 1-\frac{2\alpha }{n} + O\left( \frac{1}{n^2} \right) \right) - 1\right) ^n \\= & {} \left( 1 - \frac{4 \alpha }{n} +O\left( \frac{1}{n^2} \right) \right) ^n = e^{-4 \alpha } + o(1). \end{aligned}$$

Since this holds for any fixed $\alpha >0$, bearing in mind that $\mathcal {A}$ is closed in $\mathcal {P}$ (and hence the set $\mathcal {A}_{2}\subseteq [-1,1]^{2}$ is closed in the usual sense), we indeed find that the curve $(x,x^4)$ lies in the attainable set for every $x \in (0,1)$. It is easy to see that also (0, 0) and (1, 1) are attainable. By reflecting the curve $(x,x^{4})$ (for $x \ge 0$) in the *x*-axis (using that $(-1,1)$ is attainable and multiplying) we find that $(x,x^{4})$ is attainable for $x \in [-1,1]$. $\square$

## Proof of Theorem 1.6: fractal structure for $x<\frac{1}{3}$

It is obvious that the second assertion of Theorem 1.6 implies the first part, so we only need to prove the second one. However, since the proof of the second assertion is fairly complicated we give a brief outline of how the first assertion can be deduced, and then indicate how to augment the argument to give the second assertion.

We are to understand the closure of all the points (*x*, *y*) of the form

28 $$\begin{aligned} (x,y)=\prod \limits _{i=1}^{K}(G_{A_{i}}(t_{i}),G_{A_{i}}(2t_{i})) \end{aligned}$$

with $A_{i}\ge 2$ arbitrary integers. Using that $G_{A}(\pi /2+t)$ is either even or odd (depending on the parity of *A*) and that $G_{A}(2(\pi /2+t))$ is even, together with signs of *x*-coordinates being irrelevant (since (*x*, *y*) is attainable if and only if $(-x,y)$ is attainable) we may assume that $t_{i}\in \left[ 0,\frac{\pi }{2}\right]$ for all *i*. A curve $(x_{0},y_{0})=(G_{A_{0}}(t_{0}),G_{A_{0}}(2t_{0}))$ turns out to intersect the line $y=1$ with $|x|\le \frac{1}{3}$ only for $A_{0}$ odd, and further forces $t_{0}=\frac{\pi }{2}$, and $x=\pm \frac{1}{A}$. Hence the point (*x*, *y*) as in (28) satisfies $y=1$ only for $A_{i}$ odd and $t_{i}=\frac{\pi }{2}$ for all $i\le K$, whence $(x,y)=(\pm \frac{1}{A},1)$ with $A=\prod _{i=1}^{K}A_{i}$.

To prove the second assertion we investigate a (fairly large) neighborhood of the point $(\frac{1}{A},1)$; given an odd *A* we consider all finite products (28) with $A=\prod _{i=1}^{K}A_{i}$ and $t_{i}\approx \frac{\pi }{2}$ (and $A_{i} \ge 3$.) We will prove that all products (*x*, *y*) of this form will stay between two curves defined below; after taking logarithms this will amount to the fortunate log-convexity of the curves $(G_{A_{0}}(t),G_{A_{0}}(2t))$, $A_{0}\ge 3$ odd, in the suitable range (see Lemma 6.8 below). We argue that this property is invariant with respect to multiplying by curves $(G_{A_{1}}(t),G_{A_{1}}(2t))$ for $A_{1}\ge 2$ even, and also for odd $A_{1} \ge 3$ for *t* near $\pi /2$.

### Proof of the second assertion of Theorem 1.6

To prove the main result of the present section we will need the following results. (The proofs of Propositions 6.1 and 6.2 are postponed to Appendices 1 and 2, respectively.)

#### **Proposition 6.1**

Let $\{ A_{i}\}_{i}$ be a finite collection of integers $A_{i}\ge 2$, and consider a point (*x*, *y*) of the form

29 $$\begin{aligned} (x,y) = \left( \prod _{i} G_{A_{i}}(t_{i}), \prod _{i} G_{A_{i}}(2t_{i}) \right) , \end{aligned}$$

where all $t_{i} \in [0, \pi /2]$. Assume that one of the following is satisfied:

- There exists *i* such that $A_{i} \ge 3$ is odd and $t_{i} \in [\pi /(2A_{i}), \pi /2 - \pi /(2A_{i})]$.
- There exists *i* such that $A_{i}$ is even and $t_{i} \ge \pi /(2A_i)$.

Then necessarily

$$\begin{aligned} y\le (2|x|-1)^{2}. \end{aligned}$$

#### **Proposition 6.2**

Let $A\ge 3$ be an odd number, and

$$\begin{aligned} A=\prod \limits _{i=1}^{K}A_{i} \end{aligned}$$

an arbitrary (fixed) factorization of *A* into (not necessarily co-prime) integers $A_{i}\ge 3$. For $x\le \frac{1}{A}$ define

30 $$\begin{aligned} g_{\{A_{i}\}}(x) = \sup \limits _{(t_{i})_{i} \in \mathcal {X}_{\{A_{i}\}}(x)} \prod \limits _{i=1}^{K} G_{A_{i}}(2t_{i}), \end{aligned}$$

the supremum taken w.r.t. all $(t_{i})_{i\le K}$ lying in

31 $$\begin{aligned} \mathcal {X}_{\{A_{i}\}}(x):= \left\{ (t_{i})_{i}:\forall i\le K, \, t_{i}\in \left[ \frac{\pi }{2}-\frac{\pi }{2A_{i}},\pi /2\right] , \, \left| \prod \limits _{i=1}^{K}G_{A_{i}}(t_{i})\right| = x \right\} . \end{aligned}$$

Then for every $0<x < \frac{1}{A}$ there exists an index $i_{0}=i_{0}(x)\le K$ and $t\in [\frac{\pi }{2}-\frac{\pi }{2A_{i_{0}}},\pi /2]$ such that^3^

$$\begin{aligned} (x,g_{\{A_{i}\}}(x)) = \left( \frac{A_{i_{0}}}{A}|G_{A_{i_{0}}}(t)|,G_{A_{i_{0}}}(2t)\right) , \end{aligned}$$

and moreover the map $x\mapsto i_{0}(x)$ is piecewise constant. In particular, the function $g_{\{A_{i}\}}(x)$ is continuous, analytic in some (left) neighbourhood of $x=\frac{1}{A}$, and piecewise analytic on $(0,\frac{1}{A}]$.

We may finally define the function $f_{2;k}$ introduced in Theorem 1.6.

#### **Definition 6.3**

Given $$k\ge 1$$, $$0<x \le \frac{1}{2k+1}$$, define

$$\begin{aligned} f_{2;k}(x)=\max \limits _{\prod \limits _{i=1}^{K}A_{i}=2k+1} g_{\{A_{i}\}}(x), \end{aligned}$$

the maximum taken w.r.t. all non-trivial factorizations of $2k+1$, i.e., all sets of (odd) integers $\{ A_{i}\}_{i=1}^{K}\subseteq {\mathbb Z}_{\ge 3}$, whose product is $2k+1$.

#### *Remark 6.4*

1. For $A\ge 3$ odd, $t\in \left[ \frac{\pi }{2}-\frac{\pi }{2A},\pi /2\right]$ we have
  32 $$\begin{aligned} |G_{A}(t)|\le \frac{1}{A}. \end{aligned}$$
2. By the definition of $g_{\{A_{i}\}}$ and $f_{2;k}$, if (*x*, *y*) is of the form
  $$\begin{aligned} (x,y)=\prod \limits _{i=1}^{K}(|G_{A_{i}}(t_{i})|,G_{A_{i}}(2t_{i})) \end{aligned}$$
  with all $A_{i}\ge 3$ odd, $x>0$, and if in addition for all *i* we have
  $$\begin{aligned} t_{i}\in \left[ \frac{\pi }{2}-\frac{\pi }{2A_{i}},\pi /2\right] \end{aligned}$$
  (whence $x\le \frac{1}{2k+1}$ via (32)), then necessarily
  33 $$\begin{aligned} y\le g_{\{A_{i}\}_{i\le K}}(x) \le f_{2;k}(x), \end{aligned}$$
  where *k* is defined as in
  $$\begin{aligned} \prod \limits _{i=1}^{K}A_{i}=2k+1. \end{aligned}$$
3. Proposition 6.2 implies that for $k\ge 1$ and $x<\frac{1}{2k+1}$,
  $$\begin{aligned} f_{2;k}(x)=\max \limits _{1<A | 2k+1 }\max \limits _{\left\{ t\in \left[ \frac{\pi }{2}-\frac{\pi }{2A},\pi /2\right] : \left| \frac{A}{2k+1}G_{A}(t)\right| =x\right\} } G_{A}(2t), \end{aligned}$$
  a maximum w.r.t. all (odd) divisors $A>1$ of $2k+1$; the latter yields an algorithm for computing $f_{2;k}(x)$, reducing the original problem into maximizing a finite set of numbers.

The following 3 results will be proven in Appendix 2.

#### **Lemma 6.5**

Let $A\ge 3$ be an odd integer, and $\eta _{A}$ be the parametric curve in ${\mathbb R}^{2}$ defined by

34 $$\begin{aligned} \eta _{A}(t) = (\eta _{A;1}(t),\eta _{A;2}(t))=(\log (A\cdot |G_{A}(t)|),\log (G_{A}(2t))), \end{aligned}$$

for $t\in (\frac{\pi }{2}-\frac{\pi }{2A},\frac{\pi }{2}]$. Then we may re-parameterize $\eta$ as $(z,h_{A}(z))$ for some analytic function $h:(-\infty ,0)\rightarrow {\mathbb R}_{\le 0}$ with $h(0)=0$, and moreover $0< h'(z) \le \frac{4}{3}$ everywhere in the above range.

#### **Corollary 6.6**

Let $\{ A_{i}\}_{i=1}^{K}\subseteq {\mathbb Z}_{\ge 3}$ be a set of odd integers, $A=\prod _{i=1}^{K}A_{i}$, and (*x*, *y*) of the form

$$\begin{aligned} (x,y) = \prod \limits _{i=1}^{K}(G_{A_{i}}(t_{i}),G_{A_{i}}(2t_{i})), \end{aligned}$$

such that for all $i\le K$ we have $t_{i}\in [\frac{\pi }{2}-\frac{\pi }{2A_{i}},\frac{\pi }{2}]$. Then necessarily

$$\begin{aligned} y\ge (Ax)^{4/3} . \end{aligned}$$

#### **Lemma 6.7**

For every $x_{1},x_{2} \in [0,1]$ the following inequality holds:

35 $$\begin{aligned} (2x_{1}^{2}-1)\cdot (2x_{2}^{2}-1) \ge (2(x_{1}x_{2})^{2}-1). \end{aligned}$$

We are finally in a position to prove Theorem 1.6 (with the first assertion following from the second.)

#### *Proof of the second assertion of Theorem 1.6 assuming the results above*

   We first prove that any point $(x,y)\in \mathcal {A}_{2}$ with $0<x\le \frac{1}{3}$ either satisfies $y\le (2x-1)^{2}$ or $(x,y)\in \mathcal {D}_{0,x_{k}}(f_{1;k},f_{2;k})$ for some $k\ge 1$, i.e. establish the inclusion $\subseteq$ of (7). Since $\mathcal {A}_{2}$ is the closure (in ${\mathbb R}^{2}$) of the set of finite products

36 $$\begin{aligned} (x,y)=\prod \limits _{i=1}^{K}(G_{A_{i}}(t_{i}),G_{A_{i}}(2t_{i})), \end{aligned}$$

with some $A_{i}\ge 2$, $t_{i}\in [0,\pi ]$, and the set on the r.h.s. of (7) is closed in $\{x>0\}$, it is sufficient to prove it for the finite products (36).

Thus let (*x*, *y*) be given by a finite product (36); by the invariance of $\mathcal {A}_{2}$ w.r.t. $x\mapsto -x$ we may assume that all $t_{i}$, $i\le K$ satisfy $t_{i}\in [0,\pi /2]$. If there exists either an odd $A_{i}$ such that $t_{i}\in [\frac{\pi }{2A_{i}}, \frac{\pi }{2}-\frac{\pi }{2A_{i}}]$, or an even $A_{i}$ such that $t_{i}\in [\frac{\pi }{2A_{i}}, \frac{\pi }{2}]$, then one of the sufficient conditions of Proposition 6.1 is satisfied, implying that $y\le (2x-1)^{2}$, so that our present statement holds.

We may then assume that for all odd $A_{i}$ we have either $t_{i}\in [0,\frac{\pi }{2A_{i}})$ or $t_{i}\in (\frac{\pi }{2}-\frac{\pi }{2A_{i}},\frac{\pi }{2}]$, and for all even $A_{i}$ we have $t_{i}\in [0,\frac{\pi }{2A_{i}})$. Up to reordering the indexes, we may assume that $K=K_{1}+K_{2}$ with $K_{1} > 0$, and where all the $A_{i}$ with $i\le K_{1}$ are odd and $t_{i}\in [\frac{\pi }{2}-\frac{\pi }{2A_{i}},\frac{\pi }{2}]$, and for all $K_{1}+1\le i\le K_{2}$ we have

37 $$\begin{aligned} t_{i}\in \left[ 0,\frac{\pi }{2A_{i}} \right] , \end{aligned}$$

whether the corresponding $A_{i}$ is odd or even. Let

38 $$\begin{aligned} A=\prod \limits _{i=1}^{K_{1}}A_{i}=2k+1. \end{aligned}$$

be the product of the first $K_{1}$ odd $A_{i}$. We claim that, with *k* as defined in (38), necessarily

39 $$\begin{aligned} f_{1;k}(x) \le y\le f_{2;k}(x). \end{aligned}$$

Define

$$\begin{aligned} (x_{0},y_{0})= \prod \limits _{i=1}^{K_{1}}(G_{A_{i}}(t_{i}),G_{A_{i}}(2t_{i})) \end{aligned}$$

and

40 $$\begin{aligned} (x_{1},y_{1})= \prod \limits _{i=K_{1}+1}^{K_{1}+K_{2}}(G_{A_{i}}(t_{i}),G_{A_{i}}(2t_{i})), \end{aligned}$$

so that

41 $$\begin{aligned} (x,y)=(x_{0},y_{0})\cdot (x_{1},y_{1}). \end{aligned}$$

Since by the above, $(x_{0},y_{0})$ satisfies the assumptions of (33), we have $y_{0}\le g_{\{A_{i}\}_{i\le K_{1}}}(x_{0})$, and by Proposition 6.2 there exists $i_{0} \le K_{1}$ and $t_{0}\in (\frac{\pi }{2}-\frac{\pi }{2A_{i_{0}}},\frac{\pi }{2}]$, so that

42 $$\begin{aligned} x_{0}=\frac{A_{i_{0}}}{A}|G_{A_{i_{0}}}(t_{0})| \end{aligned}$$

and $g_{\{A_{i}\}_{i\le K_{1}}}(x_{0}) = G_{A_{i_{0}}}(2t_{0})$; we then have

43 $$\begin{aligned} y_{0} \le G_{A_{i_{0}}}(2t_{0}). \end{aligned}$$

For the sake of brevity of notation we assume with no loss of generality that $i_{0}=1$, and consider the curve $\eta _{A_{1}}$ in ${\mathbb R}_{> 0}^{2}$ as in Lemma 6.5; by the virtue of the latter lemma we may re-parameterize $\eta _{A_{1}}$ as $(z,h_{A_{1}}(z))$ in the range $z\in (-\infty ,0]$, and $0< h_{A_{1}}'(x) \le \frac{4}{3}$ everywhere. Hence, on noting that all the logarithms involved are *negative*, the mean value theorem gives that

44 $$\begin{aligned} h_{A_{1}}(\log (Ax_{0}x_{1}))= & {} h_{A_{1}}(\log (Ax_{0})+\log (x_{1})) \nonumber \\\ge & {} h_{A_{1}}(\log (Ax_{0}))+\frac{4}{3}\log (x_{1}). \end{aligned}$$

Note that by (42) and the definition of $h_{A_{1}}$ as a re-parametrization of (34), we have

$$\begin{aligned} h_{A_{1}}(\log (Ax_{0})) = h_{A_{1}}(\log (A_{1}|G_{A_{1}}(t_{0})|) = \log G_{A_{1}}(2t_{0}) \end{aligned}$$

(recall that we assumed that $i_{0}=1$).

Substituting the latter into (44) implies that there exist a number $\theta _{1}\in \left( \frac{\pi }{2}-\frac{\pi }{2A_{1}},\frac{\pi }{2}\right]$ satisfying $A_{1}|G_{A_{1}}(\theta _{1})|=Ax_{0}x_{1}$ (note that $x_{0} \in (0,1/A]$) and

$$\begin{aligned} \log (G_{A_{1}}(2\theta _{1})) \ge \log G_{A_{1}}(2t_{0})+\frac{4}{3}\log (x_{1}). \end{aligned}$$

Equivalently,

45 $$\begin{aligned} |G_{A_{1}}(\theta _{1})|=\frac{A}{A_{1}} x_{0}x_{1} \end{aligned}$$

and

46 $$\begin{aligned} G_{A_{1}}(2\theta _{1}) \ge G_{A_{1}}(2t_{0})\cdot x_{1}^{4/3} \ge y_{0}\cdot x_{1}^{4/3}, \end{aligned}$$

by (43).

Note that for the choice $t_{1}=\theta _{1}$ and $t_{i}=\frac{\pi }{2}$ for $2 \le i \le K_{1}$, we have

47 $$\begin{aligned} \left| \prod \limits _{i=1}^{K_{1}}G_{A_{i}}(t_{i}) \right| = \frac{A}{A_{1}} x_{0}x_{1}\cdot \prod \limits _{i=2}^{K_{1}}\frac{1}{A_{i}} = x_{0}x_{1}, \end{aligned}$$

by (45) and (38). Now, bearing in mind (41), as $g_{\{A_{i}\}_{i\le K_{1}}}(x)$ is defined to be the supremum of all the expressions (30) with $\{t_{i}\}_{i\le K_{1}}$ satisfying (47), and recalling Definition 6.3 of $f_{2;k}(x)$, (46) implies that

48 $$\begin{aligned} f_{2;k}(x) \ge g_{\{A_{i}\}_{i\le K_{1}}}(x)\ge y_{0}\cdot x_{1}^{4/3}. \end{aligned}$$

On the other hand, (37) implies that for every $K_{1}+1\le i\le K_{1}+K_{2}$ we have $G_{A_{i}}(t_{i})> \frac{1}{3}$ (for *A* fixed, $G_{A}(t)$ is decreasing for $t \in [0, \pi /A]$ and it is enough to show that $G_{A}(\pi /(2A)) = (A \sin (\pi /(2A)))^{-1} > 1/3$; this in turn follows from $\sin (x)/x$ being decreasing on $[0,\pi ]$.) Hence Proposition 5.5 is applicable for each of the terms on the r.h.s. of (40), and therefore their product $(x_{1},y_{1})$ satisfies

49 $$\begin{aligned} y_{1}\le x_{1}^{4}. \end{aligned}$$

The inequality (49) together with (48) and the fact that $x^{4/3} > x^{4}$ for $x < 1$ yield that

$$\begin{aligned} f_{2;k}(x) \ge y_{0}\cdot x_{1}^{4/3} \ge y_{0}\cdot x_{1}^{4} \ge y_{0}\cdot y_{1} = y, \end{aligned}$$

as in (41), which is the second inequality of (39).

To prove the first inequality of (39) we use Corollary 6.6 to yield $y_{0} \ge (Ax_{0})^{4/3}$ with *A* as in (38). These combined imply

$$\begin{aligned} y= & {} y_{0}\cdot y_{1} \ge (Ax_{0})^{4/3}\cdot (2x_{1}^{2}-1) \ge (Ax_{0})^{4}\cdot (2x_{1}^{2}-1) \\\ge & {} (2(Ax_{0})^{2}-1) \cdot (2x_{1}^{2}-1) \end{aligned}$$

where we used the obvious inequality $x^{4}\ge 2x^{2}-1$, valid on $[-1,1]$. Finally, an application of the inequality (35) of Lemma 6.7 yields

$$\begin{aligned} y\ge 2(Ax_{0}x_{1})^{2}-1 = 2A^{2}\cdot x^{2}-1 = f_{1;k}(x), \end{aligned}$$

by the definition (8) of $f_{1;k}$, and recalling that $x_{k}=\frac{1}{2k+1}$.

Conversely, we need to prove that any point (*x*, *y*) satisfying

$$\begin{aligned} f_{1;k}(x)\le y\le f_{2;k}(x) \end{aligned}$$

necessarily lies in $\mathcal {A}_{2}$. To this end fix a number $k\ge 1$ and consider all the points (*x*, *y*) of the form

50 $$\begin{aligned} (x,y) = (s,f_{2;k}(s)) \cdot (t,2t^{2}-1) \end{aligned}$$

with $s\in (0,\frac{1}{2k+1}]$, $t\in (0,1]$ (recalling the notation (22) for componentwise multiplication). Note that by the multiplicativity of $\mathcal {A}_{2}$ (Proposition 1.2) all the points of the form (50) are attainable, i.e., $(x,y)\in \mathcal {A}_{2}$. Since $f_{2;k} (\frac{1}{2k+1})=1$, for $s=\frac{1}{2k+1}$ fixed, *t* varying in (0, 1], (*x*, *y*) attains all the curve $(x,y)=(x,f_{1;k}(x))$; for $t=1$ fixed, *s* varying in $(0,\frac{1}{2k+1})$, (*x*, *y*) attains the curve $(x,y)=(x,f_{2;k}(x))$.

We claim that for every (*x*, *y*) with $f_{1;k}(x)\le y \le f_{2;k}(x)$ there exists *s*, *t* in the range as above, satisfying (50). To show the latter statement, given such a point (*x*, *y*) consider $s\in [x,\frac{1}{2k+1}]$ and $t=\frac{x}{s}$. We are then to solve the equation

$$\begin{aligned} y=f_{2;k}(s)\cdot \left( \frac{2x^{2}}{s^{2}}-1 \right) \end{aligned}$$

for the given *y*, $s\in [x,\frac{1}{2k+1} ]$; as the r.h.s. of the latter equation attains the values $f_{1;k}(x)$ and $f_{2;k}(x)$ for $s=\frac{1}{2k+1}$ and $s=x$ respectively, we are guaranteed a solution by the intermediate value theorem. Geometrically, the above argument shows that as *s* varies, the family of parabolas

$$\begin{aligned} t\mapsto (s,f_{2;k}(s)) \cdot (t,2t^{2}-1) \end{aligned}$$

tesselates the domain $\mathcal {D}_{0,x_{k}}(f_{1;k},f_{2;k})$ (cf. the proof of Proposition 5.2 in Sect. 5.6). $\square$

### Proof of Proposition 6.2 by convexity

The convexity of the component-wise logarithm of a curve implies that finite products of points lying on that curve would stay below it. We aim at eventually proving that all the curves $\gamma _{A}=(G_{A}(t),G_{A}(2t))$, $A\ge 3$ odd, $t\in \left[ \frac{\pi }{2}-\frac{\pi }{2A},\frac{\pi }{2}\right]$, satisfy the above property (see Lemma 6.8 below). We exploit their convexity in Lemma 6.9, which, after taking logarithm, is equivalent to the statement of Proposition 6.2 (see the proof of Proposition 6.2 below); the latter follow from finite products of points on a curve, with the property above, staying below that curve.

#### **Lemma 6.8**

Let $\eta _{A}$ be the curve

$$\begin{aligned} \eta _{A}(t) = (\log (A\cdot |G_{A}(t)|),\log (G_{A}(2t))), \end{aligned}$$

$t\in (\frac{\pi }{2}-\frac{\pi }{2A},\frac{\pi }{2}]$ with $A\ge 3$ odd. Then in the above domain of *t* both components of $\eta _{A}=(\eta _{A;1},\eta _{A;2})$ are strictly increasing, and moreover $\eta _{A}$ may be re-parametrized as $(z,h_{A}(z))$ with $h_{A}:(-\infty ,0]\rightarrow {\mathbb R}$ convex analytic, increasing, and $h(0)=0$.

The somewhat technical proof of Lemma 6.8 is postponed to Appendix 2.

#### **Lemma 6.9**

Let $\{h_{i}:(-\infty ,0]\rightarrow {\mathbb R}\}_{i\le K}$ be a finite collection of continuous convex functions such that for all $i\le K$ we have $h_{i}(0)=0$. Define $h:(-\infty ,0]\rightarrow {\mathbb R}$ by

51 $$\begin{aligned} h(z)=\sup \limits _{z_{i}\le 0:\, \sum \nolimits _{i=1}^{K}z_{i}=z} \left\{ \sum \limits _{i=1}^{K}h_{i}(z_{i}) \right\} . \end{aligned}$$

Then for every $z\in (-\infty ,0]$ there exists an index $i_{0}=i_{0}(z)$ so that $h(z)=h_{i_{0}}(z).$

Before giving a proof for Lemma 6.9 we may finally prove Proposition 6.2.

#### *Proof of Proposition 6.2 assuming Lemmas 6.8 and 6.9*

Let $A=2k+1\ge 3$ be odd, and (38) be an arbitrary factorization of *A* into integers $A_{i}\ge 3$. Consider the curves $\{\eta _{A_{i}}(t):\, t\in [\frac{\pi }{2}-\frac{\pi }{2A_{i}},\frac{\pi }{2} ] \}_{i\le K}$ as defined in (34). By Lemma 6.8 all of the $\eta _{A_{i}}$ can be re-parametrized as $(z_{i},h_{A_{i}}(z_{i}))$ on $(-\infty ,0]$, with $h_{A_{i}}$ convex analytic, and $h(0)=0$.

Hence, by Lemma 6.9 for every $z\in (0,\frac{1}{A}]$ there exists $i_{0}=i_{0}(x)$, so that

$$\begin{aligned} h(z):=\sup \limits _{z_{i}\le 0:\, \sum \nolimits _{i=1}^{K}z_{i}=z} \left\{ \sum \limits _{i=1}^{K}h_{A_{i}}(z_{i}) \right\} = h_{A_{i_{0}}}(z), \end{aligned}$$

Note that, after taking logarithms, maximizing $\prod _{i=1}^{K}G_{A}(2t_{i})$ under the constraint $(t_{i})_{i\le K} \in \mathcal {X}_{\{A_{i}\}}(x)$ with $\mathcal {X}_{\{A_{i}\}}(x)$ as in (31), $0<x\le \frac{1}{A}$ is equivalent to maximizing

$$\begin{aligned} \sum \limits _{i=1}^{K}\log G_{A}(2t_{i}) = \sum \limits _{i=1}^{K}h_{A_{i}}(z_{i}) \end{aligned}$$

under the constraint $\sum _{i=1}^{K}z_{i}=z$, where $z=\log {Ax} \in (-\infty , 0]$. More formally, recalling the definition (34) of $\eta _{A_{i}}$ and $(z_{i},h_{A_{i}}(z_{i}))$ being a re-parametrization of $\eta _{A_{i}}$, the function *h*(*z*) defined as in (51), on noting that $z = \log Ax$, satisfies

52 $$\begin{aligned} h(\log (Ax)) = \log {\sup \limits _{(t_{i})_{i\le K}\in \mathcal {Y}_{\{A_{i}\}}(x)} \left\{ \prod \limits _{i=1}^{K}G_{A_{i}}(2t_{i}) \right\} }, \end{aligned}$$

where

$$\begin{aligned} \mathcal {Y}_{\{A_{i}\}}(x) = \left\{ (t_{i})_{i\le K}:\, \forall i. t_{i}\in \left[ \frac{\pi }{2}-\frac{\pi }{2A_{i}},\frac{\pi }{2}\right] , \,\sum \limits _{i=1}^{K}\log (A_{i}|G_{A_{i}}(t_{i})|) =\log (Ax)\right\} . \end{aligned}$$

Since $\sum _{i=1}^{K}\log (A_{i}|G_{A_{i}}(t_{i})|) =\log (Ax)$ is equivalent to $\sum _{i=1}^{K}\log (|G_{A_{i}}(t_{i})|) =\log (x)$ via (38), we have $\mathcal {Y}_{\{A_{i}\}}(x) = \mathcal {X}_{\{A_{i}\}}(x)$ (as in (31)), and hence (52) is

$$\begin{aligned} h(\log (Ax))=\log \left( g_{\{A_{i}\}}(x)\right) . \end{aligned}$$

The latter equality together with Lemma 6.9 then imply that we have

$$\begin{aligned} h_{i_{0}}(\log (Ax))=\log \left( g_{\{A_{i}\}_{i\le K}}(x)\right) \end{aligned}$$

for some $i_{0}\le K$; since $h_{i_{0}}$ is a re-parametrization of $\eta _{A_{i_{0}}}$, this is equivalent to

$$\begin{aligned} (\log (A_{i_{0}}|G_{A_{i_{0}}}(t_{i_{0}})|),\log (G_{A_{i_{0}}}(2t_{i_{0}}))) = (\log (Ax),\log g_{\{A_{i}\}_{i\le K}}(x)) \end{aligned}$$

for some $$t_{i_{0}}\in [\frac{\pi }{2}-\frac{\pi }{2A_{i_{0}}},\frac{\pi }{2}]$$, i.e.

$$\begin{aligned} \left( \frac{A_{i_{0}}}{A}|G_{A_{i_{0}}}(t_{i_{0}})|,G_{A_{i_{0}}}(2t_{i_{0}})\right) = (x, g_{\{A_{i}\}_{i\le K}}(x)), \end{aligned}$$

which is the first statement of the present proposition, at least for $x>0$. For $x=0$ it is sufficient to notice that for all $i\le K$,

$$\begin{aligned} (G_{A_{i}}(t), G_{A_{i}}(2t))|_{t=\frac{\pi }{2}-\frac{\pi }{2A_{i}}} = (0,0), \end{aligned}$$

so that in particular $g_{\{A_{i}\}_{i\le K}}(x) = 0$, whatever $\{A_{i}\}_{i\le K}$ are.

To see that the map $x\mapsto i_{0}(x)$ is in fact piecewise constant on $[0,\frac{1}{A}]$ (with finitely many pieces), we note that it is readily shown that on $(0, \frac{1}{A}]$, $g_{\{ A_{i}\}_{i\le K}}$ is a maximum of finitely many analytic curves (namely, $(\frac{A_{i}}{A}|G_{A_{i}}(t)|,G_{A_{i}}(2t))$), and vanishes at 0, which happens to lye on all of them. Since such a collection of analytic curves may only intersect in finitely many points for $x\in [0, \frac{1}{A}]$, it follows that $i_{0}(x)$ is uniquely determined as the maximum of these outside of finitely many points (that include (0, 0)), and $i_{0}$ is constant between any two such consecutive points. $\square$

#### *Proof of Lemma 6.9*

It is easy to check that with the assumptions of the present lemma, the function $H:(-\infty ,0]^{K}\rightarrow {\mathbb R}$ defined by

$$\begin{aligned} H(t_{1},\ldots , t_{K}) = \sum \limits _{i=1}^{K}h_{i}(t_{i}) \end{aligned}$$

is a convex function. Now fix $t<0$ and consider the set

$$\begin{aligned} \Omega (t):=\left\{ (t_{i})_{i\le K}:\, \sum \limits _{i=1}^{K}t_{i}=t, \,\,t_{i} \le 0\,\, \text {for}\,\, 1 \le i \le K \right\} \subseteq (-\infty ,0]^{K}; \end{aligned}$$

$\Omega (t)$ is a compact convex domain, and it is evident that

$$\begin{aligned} h(t)=\max \limits _{(t_{i})\in \Omega (t)}H(t_{1},\ldots , t_{k}). \end{aligned}$$

Now, a convex function cannot attain a maximum in the interior of a convex domain (all the local extrema of a convex function are necessarily minima). Hence there exists an index $i_{1}\le K$ so that

$$\begin{aligned} h(t)=\sum \limits _{i=1}^{K}h_{i}(t_{i}) \end{aligned}$$

for some $(t_{i})\in \Omega (t)$ with $t_{i_{1}}=0$, i.e. one of the elements of $(t_{i})$ must vanish. By induction, we find that all but one element of $(t_{i})$ vanish, say $t_{i}=0$ for $i\ne i_{0}$, whence $t_{i_{0}}=t$, and $h(t)=h_{i_{0}}(t)$, as $h_{i}(0)=0$ for $i\ne i_{0}$ by the assumptions of the present lemma. $\square$

## Proof of Theorem 1.4: square-free attainable measures

### *Proof*

Recall that we de-symmetrized all the probability measures by an analogue of (10). First we show that (4) holds for any square-free attainable measure; as the first inequality in (4) holds for every probability measure (13) it only remains to show that every point $(x,y) = (\hat{\mu }(1), \hat{\mu }(2))$ corresponding to a square-free attainable $\mu$ satisfies (21).

By the definition of square-free attainable measures, if $\mu$ is square-free attainable then (*x*, *y*) is lying in the closure of the set of finite products

53 $$\begin{aligned} (\tilde{x},\tilde{y})= & {} \left\{ \prod \limits _{i=1}^{K}(\cos (\theta _{i}),\cos (2\theta _{i})):\, \theta _{i}\in [0,\pi ] \right\} \nonumber \\= & {} \left\{ \prod \limits _{i=1}^{K}(x_{i},y_{i}):\, x_{i}\in [-1,1] \right\} , \end{aligned}$$

where for all $i\le K$, $y_{i} = 2x_{i}^{2}-1$. Now if $\tilde{y} > 0$ and $y_{i_{0}} < 0$ for some $i_{0}\le K$, then $(\tilde{x},\tilde{y})\in \mathcal {A}_{2}^{-}$ is a mixed sign attainable point, and (upon recalling Notation 5.6) Lemma 5.7 implies that $(\tilde{x},\tilde{y}) \in B_{1}$, i.e., $|\tilde{x}| \le 1/2$ and $\tilde{y} \le (2|\tilde{x}|-1)^{2}$.

If $\tilde{y} > 0$ and $y_{i} \ge 0$ for all *i*, then $y_{i} =2x_{i}^{2}-1 \le x_{i}^{4}$ for all *i* as it is easy to check the latter inequality explicitly, consequently $\tilde{y} \le \tilde{x}^{4}$. Since (21) holds on the collection of all products (53), it also holds on its closure, namely for square-free attainable measures. This concludes the proof of the necessity of the inequality (4).

It then remains to show the sufficiency, i.e. any point (*x*, *y*) satisfying (4) corresponds to a square-free attainable measure. We claim that the attainable measures constructed by Proposition 5.2 are in fact square-free attainable. To this end recall that the collection of all square-free attainable measures is closed under convolutions, so that the products of points corresponding to square-free attainable measures correspond to square-free attainable measures. It is then crucial to notice that the measures corresponding to points lying on the curves

$$\begin{aligned} \{(x,x^{4}):x\in [0,1]\} \end{aligned}$$

(constructed by Lemma 5.13), and

$$\begin{aligned} \{(x,(2x-1)^{2}): x\in [0,1] \} \end{aligned}$$

(a re-parameterized product of the parabola $y=2x^{2}-1$ by itself) exploited in the course of the proof of Proposition 5.2 are both square-free attainable. Hence the tessellation argument used in the proof of Proposition 5.2 works in this case too.

## Appendix 1: Proof of Proposition 6.1: below the “mixed signs” curve $y=(2x-1)^{2}$

By the assumptions of Proposition 6.1 there exists *i* such that $t_{i} \in [\pi /(2A_{i}), \pi /2-\pi /(2A_{i})]$ (for $A_{i}$ odd), or $t_{i} \in [\pi /(2A_{i}), \pi /2]$ (for $A_{i}$ even.) The following lemma exploits this property to yield more information about (at least) one point in the product.

### **Lemma 8.1**

Let $A\ge 3$ and $(x,y)=(G_{A}(t),G_{A}(2t))$. If *A* is odd and $t \in [\frac{\pi }{2A},\frac{\pi }{2}-\frac{\pi }{2A}]$, or *A* is even and $t \in [\frac{\pi }{2A},\frac{\pi }{2}]$, then either $y\le 0$, or $y\le (2|x|-1)^{2}$ and $|x|<\frac{1}{3}$.

If $A=2$ and $t \in \left[ \frac{\pi }{4},\frac{\pi }{2}\right]$, then $y=G_{2}(2t) \le 0$.

### *Proof of Proposition 6.1 assuming Lemma 8.1*

Assume with no loss of generality that the postulated index is $i=1$, i.e.

$$\begin{aligned} (x_{1},y_{1})=(G_{A_{1}}(t_{1}),G_{A_{1}}(2t_{1})) \end{aligned}$$

with either $A_{1} \ge 3$ being odd and $t \in [\frac{\pi }{2A_{1}},\frac{\pi }{2}-\frac{\pi }{2A_{1}}]$, or $A_{1}\ge 2$ being even and $t \in [\frac{\pi }{2A_{1}},\frac{\pi }{2}]$. Suppose first that $y_{1}\le 0$. In this case the point (*x*, *y*) is “mixed sign attainable” (cf. Definition 5.3), so that Lemma 5.4 implies that $y\le (2|x|-1)^{2}$.

Otherwise we assume that $y_{1}>0$ and $y>0$. Then Lemma 8.1 implies that $A\ge 3$, and $|x_{1}|<\frac{1}{3}$, whence

$$\begin{aligned} 0<y\le y_{1} \le (2|x_{1}|-1)^{2} \le (2|x|-1)^{2}, \end{aligned}$$

since $|x|\le |x_{1}|$ and the function $x\mapsto (2x-1)^{2}$ is decreasing on $\left[ 0,\frac{1}{2}\right]$. $\square$

### *Proof of Lemma 8.1*

First, upon recalling that for $A=2$ we have $G_{2}(t)=\cos (t)$, the second statement of Lemma 8.1 is obvious. We are left with proving the first statement. For $A=3$ if $t \in \left[ \frac{\pi }{6},\frac{\pi }{3}\right]$, then

$$\begin{aligned} y=\frac{\sin (6t)}{3\sin (2t)} \le 0 \end{aligned}$$

again. We may thus assume that $A\ge 4$.

Next, we would like to consolidate the even and the odd *A* cases, by showing that if *A* is even and $t\in [\frac{\pi }{2}-\frac{\pi }{2A}, \frac{\pi }{2}]$, then the statement of the present lemma holds. To do this we note that in this range $2At\in [(A-1)\pi ,A\pi ]$, so that

$$\begin{aligned} G_{A}(2t)=\frac{\sin (2At)}{A\sin (2t)}\le 0 \end{aligned}$$

once more.

Hence we may assume that $t\in [\frac{\pi }{2A}, \frac{\pi }{2}- \frac{\pi }{2A}]$, whether *A* is even or odd. We would like to further cut out the short intervals $[\frac{\pi }{2A}, \frac{\pi }{A}]$ and $[\frac{\pi }{2}-\frac{\pi }{A}, \frac{\pi }{2}-\frac{\pi }{2A}]$, i.e. establish the validity of the present lemma in these intervals. If $t\in [\frac{\pi }{2A}, \frac{\pi }{A} ]$ whether *A* is even or odd, then $2At\in [\pi ,2\pi ]$, so that $y=G_{A}(2t) \le 0$ in this regime too.

If $t\in [\frac{\pi }{2}-\frac{\pi }{A}, \frac{\pi }{2}-\frac{\pi }{2A} ]$, then $2At\in [(A-2)\pi ,(A-1)\pi ]$, so that if *A* is odd then $y=G_{A}(2t)=\frac{\sin (2At)}{A\sin (2t)}\le 0$. In the remaining case *A* even, for the same range $t\in [\frac{\pi }{2}-\frac{\pi }{A}, \frac{\pi }{2}-\frac{\pi }{2A} ]$, we write $A=2B$ for $B\in {\mathbb Z}$, and note that

$$\begin{aligned} (x,y)= & {} (G_{A}(t),G_{A}(2t)) = \left( \frac{\sin (Bt)\cos (Bt)}{B\sin (t)},\frac{\sin (2Bt)\cos (2Bt)}{B\sin (2t)}\right) \\= & {} (G_{B}(t),G_{B}(2t))\cdot (G_{2}(t),G_{2}(2t)). \end{aligned}$$

Hence if in turn *B* is even, then $G_{B}(2t) = \frac{\sin (2Bt)}{B\sin (2t)}\le 0$, since $2Bt\in [(B-1)\pi ,(B-1)\pi +\frac{\pi }{2}].$ Hence (*x*, *y*) is mixed sign attainable, and therefore by Lemma 5.4, $y\le (2|x|-1)^{2}$, and, in addition, $|x|\le \frac{1}{3}$ by Lemma 5.11.

Otherwise, if *B* is odd, we may assume that $A\ge 6$ is even (in the same range $t\in [\frac{\pi }{2}-\frac{\pi }{A}, \frac{\pi }{2}-\frac{\pi }{2A} ]$); in this case we claim that $|x|=|G_{A}(t)| \le \frac{1}{5}$ and $y= |G_{A}(2t)| \le \frac{1}{3}$. As $\frac{1}{3}\le (2/5-1)^{2}$, and $x\mapsto (2x-1)^{2}$ is decreasing on $[0,\frac{1}{2}]$ this is sufficient to yield $y\le (2|x|-1)^{2}$. To show this, we first note that $G_{A}(2t)=\pm G_{A}(2(\pi /2-t))$; hence Lemma 5.11 implies that $y\le \frac{1}{3}$ indeed. Concerning the value of |*x*|, we have for *t* in the range as above (bearing in mind that $A\ge 6$):

$$\begin{aligned} |G_{A}(t)|\le & {} \frac{1}{A\sin (t)}\le \frac{1}{A\sin (\pi /2-\pi /A)} = \frac{1}{A\cos (\pi /A)} \\\le & {} \frac{1}{6\cos (\pi /6)} = 0.19\ldots < \frac{1}{5}, \end{aligned}$$

since $A\mapsto A\cdot \cos (\pi /A)$ is strictly increasing for $A \ge 6$.

Finally, we take care of the case $A\ge 4$, whether *A* is even or odd, and the remaining range

54 $$\begin{aligned} t\in \left[ \frac{\pi }{A},\frac{\pi }{2}-\frac{\pi }{A}\right] , \end{aligned}$$

and $(x,y)=(G_{A}(t),G_{A}(2t))$. Noting that $\sin (t)\ge \frac{2}{\pi }t$ everywhere on $[0,\frac{\pi }{2}]$, we find that for $t\in [\frac{2\pi }{A},\frac{\pi }{2}]$,

$$\begin{aligned} |G_{A}(t)|\le \frac{1}{A\sin (t)} \le \frac{\pi }{2}\frac{1}{A\cdot 2\pi /A} = \frac{1}{4}. \end{aligned}$$

Hence (under the assumption (54) on *t*), if $t>\frac{2\pi }{A}$, $|x|=|G_{A}(t)| \le \frac{1}{4}$, and (using the natural symmetry $G_{A}(t)=\pm G_{A}(\pi -t)$), $y\le |y| = |G_{A}(2t)| \le \frac{1}{4}$.

If both $|x|\le \frac{1}{4}$ and $y\le \frac{1}{4}$, then $y\le (2|x|-1)^{2}$, as $x\mapsto (2x-1)^{2}$ is decreasing on $[0,\frac{1}{2}]$. Hence we are left with taking care of the range $t\in [\frac{\pi }{A},\frac{2\pi }{A}]$, where we still have $y\le \frac{1}{4}$, and we may assume $x>\frac{1}{4}$. Moreover, if $t\in [\frac{3\pi }{2A},\frac{2\pi }{A}]$, $2At\in [3\pi ,4\pi ]$, so that $y=G_{A}(2t) \le 0$, hence it is enough to prove the statement for $t\in [\frac{\pi }{A},\frac{3\pi }{2A}]$.

Now, recall that by Lemma 5.10 the function $t\mapsto \frac{\sin {t}}{t}$ is decreasing on $[0,\pi ]$, so that, bearing in mind that $A\ge 4$,

$$\begin{aligned} \frac{\sin {t}}{t}\ge \frac{\sin (At/4)}{At/4}, \end{aligned}$$

and thus

55 $$\begin{aligned} |x|= & {} |G_{A}(t)| = \frac{|\sin (At)|/(At)}{|\sin (t)|/t} \le \frac{|\sin (At)|/(At)}{\sin (At/4)/(At/4)} \nonumber \\= & {} \frac{|\sin (At)|}{4\sin (At/4)} = |G_{4}(s)| =:|x'|, \end{aligned}$$

where we rescale by letting $s = \frac{At}{4} \in [\frac{\pi }{4},\frac{3\pi }{8}]$. Arguing along the same lines we obtain

56 $$\begin{aligned} |y|=|G_{A}(2t)|\le |G_{4}(2s)| =: |y'| \end{aligned}$$

(note that $2At/4=At/2 <\pi$, so that Lemma 5.10 is valid in this range).

Since

$$\begin{aligned} G_{4}(s) = \frac{\sin (4s)}{4\sin (s)} = \cos (s)\cos (2s)=G_{2}(s)\cdot G_{2}(2s), \end{aligned}$$

we have that

$$\begin{aligned} (x',y')=(G_{4}(s),G_{4}(2s)) = (G_{2}(s),G_{2}(2s))\cdot (G_{2}(2s),G_{2}(4s)), \end{aligned}$$

is a product of two attainable points, and moreover, since $s\in [\frac{\pi }{4},\frac{3\pi }{8}]$, $G_{2}(2s) =\cos (2s) \le 0$ (and also $G_{2}(4s)\le 0$). That means that $(x',y')$ is “mixed sign attainable” (cf. Definition 5.3), and hence Lemma 5.4 implies that $y'\le (2|x'|-1)^2$. Finally, bearing in mind (55) and (56), as well as $x\mapsto (2x-1)^2$ decreasing on $[0,\frac{1}{2}]$, we have

$$\begin{aligned} y\le |y'| \le (2x'-1)^{2} \le (2x-1)^{2}. \end{aligned}$$

$$\square $$

## Appendix 2: Proof of auxiliary technical lemmas

### *Proof of Lemma 6.8*

First, by using some simple trigonometric identities (in particular, that $\sin (\pi /2-t) = \cos ( t)$), we may re-parametrize $\eta _{A}(t)$ as

$$\begin{aligned} \eta _{A}(t)= & {} (x(t),y(t))=\left( \log \left( A\frac{\cos (At)}{A\cos (t)}\right) , \ \log \left( \frac{\cos (At)}{\cos (t)}\cdot \frac{\sin (At)}{A\sin (t)}\right) \right) \\= & {} \bigg ( \log \cos (At)-\log (\cos (t)), \\&\times \log (\cos (At))-\log (\cos (t)) +\log (\sin (At))-\log (A\sin (t)) \bigg ), \end{aligned}$$

for $t\in [0,\frac{\pi }{2A}]$. By taking the derivatives, it is easy to see that both *x*(*t*) and *y*(*t*) are strictly decreasing, thus, by the inverse function theorem, the curve (*x*(*t*), *y*(*t*)) can be re-parametrized as $(x,h_{A}(x))$ with $h_{A}:(-\infty ,0]\rightarrow {\mathbb R}$ *real analytic* and strictly increasing. Hence to prove that $\eta _{A}$ is convex (or equivalently, that $h_{A}$ is convex), it is sufficient to show that the slope

$$\begin{aligned} \frac{dy}{dx}=\frac{y'(t)}{x'(t)} = 1+\frac{(\log (\sin (At))-\log (A\sin {t}))'}{(\log (\cos (At))-\log (\cos {t}))'} \end{aligned}$$

is decreasing on $(0,\frac{\pi }{2A})$, which in turn is equivalent to the function

$$\begin{aligned} t\mapsto \frac{(\log (\sin (At))-\log (\sin {t}))'}{(\log (\cos (At))-\log (\cos {t}))'} \end{aligned}$$

being decreasing on the same domain. We rescale by setting $s=At$ and let $\alpha :=\frac{1}{A}\in (0,\frac{1}{3}]$, $g(s):=-\log (\sin (s))$, $f(s):=-\log (\cos (s))$; we are then to prove that

$$\begin{aligned} s\mapsto \frac{(g(s)-g(\alpha s))'}{(f(s)-f(\alpha s))'} \end{aligned}$$

is decreasing on $(0,\frac{\pi }{2})$.

Recall the product expansion formulas

$$\begin{aligned} \sin (x) = x\prod \limits _{k=1}^{\infty } \left( 1-\frac{x^{2}}{k^{2}\pi ^{2}} \right) , \quad \cos (x) = \prod \limits _{k=1}^{\infty } \left( 1-\frac{4x^{2}}{(2k-1)^{2}\pi ^{2}} \right) \end{aligned}$$

of the sine and cosine respectively, and the Taylor series expansion $-\log (1-x)=\sum _{k=1}^{\infty }\frac{x^{k}}{k}.$ Under the above notation we have

$$\begin{aligned} f(s) = \sum \limits _{i=1}^{\infty } a_{i}s^{2i}, \quad \quad h(s):=g(s)+\log (s) = \sum \limits _{j=1}^{\infty } b_{j}s^{2j}, \end{aligned}$$

with

$$\begin{aligned} a_{i} = \frac{2^{2i}\zeta ^{*}(2i)}{i\pi ^{2i}}>0 ; \quad \; b_{j}=\frac{\zeta (2j)}{j\pi ^{2j}}>0, \end{aligned}$$

where $$\zeta $$ is the usual Riemann Zeta function, and $$\zeta ^{*}(r):= \sum _{k=1}^{\infty }\frac{1}{(2k-1)^{r}},$$ for $$ r>1$$.

We then have

$$\begin{aligned} F(s):= f(s)-f(\alpha s) = \sum \limits _{i=1}^{\infty }a_{i}(1-\alpha ^{2i})s^{2i}, \end{aligned}$$

and

$$\begin{aligned} G(s):= & {} g(s)+\log (s)-(g(\alpha s)+\log (\alpha s)) =g(s)-g(\alpha s)-\log (\alpha ) \\= & {} \sum \limits _{j=1}^{\infty }b_{j}(1-\alpha ^{2j})s^{2j}-\log (\alpha ). \end{aligned}$$

Further, since $(g(s)-g(\alpha s))' = G'(s)$ and $(f(s)-f(\alpha s))' = F'(s)$ it is enough to prove that

$$\begin{aligned} G''(s)F'(s)-G'(s)F''(s)<0 \end{aligned}$$

on $s\in (0,\frac{\pi }{2})$; note that $G'' \cdot F' - G' \cdot F''$ is defined and analytic on the interval $(0,\frac{\pi }{2})$. Now, we have

$$\begin{aligned} G''(s)F'(s)= & {} \sum \limits _{j=1}^{\infty }b_{j}\cdot 2j(2j-1)(1-\alpha ^{2j})s^{2j-2}\cdot \sum \limits _{i=1}^{\infty }a_{i}\cdot 2i(1-\alpha ^{2i})s^{2i-1}\\= & {} 4(1-\alpha ^{2})^{2}a_{1}b_{1}s+\sum \limits _{k=1}^{\infty }c_{k}s^{2k+1}, \end{aligned}$$

and

$$\begin{aligned} G'(s)F''(s)= & {} \sum \limits _{j=1}^{\infty }b_{j}\cdot 2j(1-\alpha ^{2j})s^{2j-1}\cdot \sum \limits _{i=1}^{\infty }a_{i}\cdot 2i(2i-1)(1-\alpha ^{2i})s^{2i-2}\\= & {} 4 (1-\alpha ^{2})^{2}a_{1}b_{1}s+\sum \limits _{k=1}^{\infty }d_{k}s^{2k+1}, \end{aligned}$$

and similarly

$$\begin{aligned} h''(s)f'(s)=\frac{1}{3}s+\sum \limits _{k=1}^{\infty }\gamma _{k}s^{2k+1} \end{aligned}$$

and

$$\begin{aligned} h'(s)f''(s)=\frac{1}{3}s+\sum \limits _{k=1}^{\infty }\delta _{k}s^{2k+1}, \end{aligned}$$

where for $k\ge 1$ we have $0<c_{k}<\gamma _{k}$, and (since $a_{i}, b_{j} \ge 0$ together with $\alpha \le 1/3$)

$$\begin{aligned} d_{k}\ge (1-\alpha ^{2})^{2} \delta _{k}>\frac{3}{4}\delta _{k}>0 \end{aligned}$$

(say).

Hence

57 $$\begin{aligned} G''(s)F'(s)-4(1-\alpha ^{2})^{2}a_{1}b_{1}s < \left( h''(s)f'(s)-\frac{1}{3}s\right) \end{aligned}$$

and

58 $$\begin{aligned} G'(s)F''(s)-4(1-\alpha ^{2})^{2}a_{1}b_{1}s > \frac{3}{4}\left( h'(s)f''(s)-\frac{1}{3}s\right) . \end{aligned}$$

In a moment we are going to show that the inequality

59 $$\begin{aligned} K(s):=\frac{h'(s)f''(s)-\frac{s}{3}}{h''(s)f'(s)-\frac{s}{3}} \ge 2 \end{aligned}$$

holds for $s\in (0,\frac{\pi }{2})$. Assuming (59), use (57) and (58) to finally obtain (note that $\gamma _{k} > 0$ for all *k* and hence $h''(s)f'(s) -s/3 >0$ for $s \in (0,\pi /2)$) that

$$\begin{aligned} G''(s)F'(s) - G'(s)F''(s)< & {} \left( h''(s)f'(s)-\frac{1}{3}s\right) - \frac{3}{4}\left( h'(s)f''(s)-\frac{1}{3}s\right) \\< & {} -\frac{1}{2}\left( h''(s)f'(s)-\frac{1}{3}s\right) <0. \end{aligned}$$

Now we turn to showing (59). We may compute explicitly *K*(*s*) to be

$$\begin{aligned} K(s)=\frac{-s\left( \cos (s)^2\cdot \sin (s)\cdot s^2+3\cdot \cos (s)\cdot s-3\cdot \sin (s)\right) }{\cos (s)\cdot \left( -\cos (s)\cdot \sin (s)\cdot s^3+3\cdot \cos (s)^2+3\cdot s^2-3\right) }, \end{aligned}$$

with both numerator and denominator non-negative; hence (59) is equivalent to

$$\begin{aligned} q(s):= & {} -s(\cos (s)^2\cdot \sin (s)\cdot s^2+3\cdot \cos (s)\cdot s-3\cdot \sin (s)) \\&- 2\cos (s)\left( -\cos (s)\cdot \sin (s)\cdot s^3+3\cdot \cos (s)^2+3\cdot s^2-3\right) \ge 0, \end{aligned}$$

and we may simplify

60 $$\begin{aligned} q(s) = s^{3}\cos (s)^{2}\sin (s)-9s^{2}\cos (s)+3s\sin (s)+6\cos (s)\sin (s)^{2} \end{aligned}$$

That $q(s)\ge 0$ on $\left[ 0,\frac{\pi }{2}\right]$ is the content of Lemma 9.1. $\square$

### **Lemma 9.1**

The function *q*(*s*), defined by (60), satisfies $q(s)\ge 0$ on $s\in \left[ 0,\frac{\pi }{2}\right]$.

### *Proof of Lemma 6.8*

The result of the lemma is evident from plotting *q*(*s*) numerically, but a formal argument can be given along the following lines. We have

$$\begin{aligned} q(s)= & {} \frac{s^{3}}{2}\cos (s)\sin (2s)-9s^{2}\cos (s)+3s\sin (s)+3\sin (2s)\sin (s)\\= & {} \frac{s^{3}}{4}(\sin (3s)+\sin (s)) -9s^{2}\cos (s)+3s\sin (s)\\&+\frac{3}{2}(\cos (s)-\cos (3s)) \end{aligned}$$

thus we may Taylor expand *q* around $s=0$ (we caution the reader that $d_{k}$ is not the same as in the proof of the previous Lemma):

61 $$\begin{aligned} q(s) = \sum \limits _{k=4}^{\infty }d_{k}s^{2k}, \end{aligned}$$

where

62 $$\begin{aligned} d_{k}= & {} (-1)^{k}\left( \frac{9}{(2k-2)!} +\frac{3^{2k-3}+1}{4\cdot (2k-3)!}\right) \nonumber \\&+(-1)^{k+1}\left( \frac{3^{2k+1}-3}{2\cdot (2k)!} +\frac{3}{(2k-1)!} \right) \end{aligned}$$

in particular $d_{4}=\frac{29}{105}$, $d_{5}=-\frac{797}{9450}$. The general formula (62) clearly implies that as $k\rightarrow \infty$, $d_{k}\sim (-1)^{k} \frac{3^{2k-3}}{4(2k-3)!},$ and moreover, a crude estimate (using that $|(3^{2k+1}-3)/(2 \cdot 2k(2k-1)(2k-2))| < 3^{2k-3} \cdot 3^{4}/(16(k-1)^{3})$ and $| 9/(2k-2) - 3/((2k-1)(2k-2))| < 9/(2k-2)$) shows that for $k \ge 4$,

$$\begin{aligned} d_{k} = (-1)^{k} \frac{3^{2k-3}}{4(2k-3)!}\left( 1+\theta \left( \frac{1}{3^{2k-7}(2k-2)} + \frac{1}{3^{2k-3}}+\frac{21}{(k-1)^3} \right) \right) , \end{aligned}$$

where^4^ $|\theta |\le 1$. For $k\ge 6$ we then have

63 $$\begin{aligned} d_{k} = (-1)^{k} \frac{3^{2k-3}}{4(2k-3)!}\left( 1+\frac{1}{5}\theta \right) ; \end{aligned}$$

it is evident that the signs of $d_{k}$ are alternating.

Now separate the summands of (61) corresponding to $k\le 5$ from the rest; the remaining summands are united into pairs, i.e. write

64 $$\begin{aligned} q(s) = s^{8}q_{0}(s)+\sum \limits _{r=3}^{\infty }\left( d_{2r}s^{4r}+d_{2r+1}s^{4r+2}\right) , \end{aligned}$$

where

$$\begin{aligned} q_{0}(s)=d_{4}+d_{5}s^{2} = \frac{29}{105} - \frac{797}{9450}s^2 \ge 0 \end{aligned}$$

on $[0,\frac{\pi }{2}]$, using the explicit Taylor coefficients mentioned above.

For the remaining terms, note that by the above, for $r\ge 3$ we have $d_{2r}>0$ and $d_{2r+1}<0$, and upon employing (63) with $k=2r$ and $k=2r+1$, we obtain

$$\begin{aligned} |d_{2r+1}|< & {} \frac{6}{5}\frac{3^{4r-1}}{4(4r-1)!} < \frac{6}{5}\cdot 9 \cdot \frac{1}{(4r-2)^{2}}\frac{3^{4r-3}}{4(4r-3)!} \\\le & {} \frac{6}{5}\cdot 9 \cdot \frac{1}{(4r-2)^{2}} \cdot \frac{5}{4}|d_{2r}| < 0.2|d_{2r}|. \end{aligned}$$

Hence each of the summands in (64), for $s\in [0,\frac{\pi }{2}]$, satisfies:

$$\begin{aligned} d_{2r}s^{4r}+d_{2r+1}s^{4r+2} > d_{2r}s^{4r}-0.2d_{2r}s^{4r+2} \ge 0 \end{aligned}$$

as $0.2 \left( \frac{\pi }{2}\right) ^{2} < 1.$ Finally $q(s)\ge 0$, since all the summands in (64) are nonnegative. $\square$

### *Proof of Lemma 6.5*

By Lemma 6.8 (note that the proof of Lemma 6.8 does not use Lemma 6.5) we may re-parametrize $\eta _{A}$ as $(x,h_{A}(x))$ on $x\in (-\infty ,0]$. Since both components $\eta _{A;1}$ and $\eta _{A;2}$ are strictly increasing, it follows that $h_{A}'(x)>0$ everywhere, and $h_{A}'(x)\le \frac{4}{3}$ follows from the convexity of $h_{A}$, and the explicit computation $h_{A}'(0)=\frac{4}{3}$. $\square$

### *Proof of Corollary 6.6*

By the multiplicativity, it is sufficient to prove the statement for a single $A_{i}$, i.e. that if

$$\begin{aligned} (x,y)=(G_{A}(t),G_{A}(2t)) \end{aligned}$$

with *A* odd and $t\in [\frac{\pi }{2}-\frac{\pi }{2A},\frac{\pi }{2}]$, then

$$\begin{aligned} y\ge (Ax)^{4/3}. \end{aligned}$$

As we may assume with no loss of generality that $x>0$ (note that $y>0$ by the assumption of $t_{i}$ being near $\pi /2$) the latter is equivalent to

65 $$\begin{aligned} \log y \ge \frac{4}{3} \log (Ax). \end{aligned}$$

Note that, with $\eta _{A}$ defined as in Lemma 6.5, $\eta _{A}(t)= (z,h_{A}(z))=(\log (Ax),\log (y))$, with $h_{A}$ analytic convex, $h_{A}(0)=0$, and a straightforward computation shows that $h_{A}'(0)=\frac{4}{3}$. By the convexity of $\eta _{A}$ then the curve lies above its tangent line at the origin, i.e. (65) follows. $\square$

### *Proof of Lemma 6.7*

The claimed inequality follows from the identity

$$\begin{aligned} (2x_{1}^{2}-1)(2x_{2}^{2}-1)-(2(x_{1}x_{2})^{2}-1) = 2(x_{1}^{2}-1)(x_{2}^{2}-1). \end{aligned}$$

$$\square $$

## Footnotes

1 One may think about this procedure as a number theoretical analogue of choosing an independent identically distributed copy of a given random variable.

2 Since $G_{A}$ is even we have $\gamma _{k;2}(-\theta ) = \gamma _{k;2}(\theta )$, and hence it is enough to consider $\theta \in [0,\pi ]$ (rather than $\theta \in [0,2\pi ]$.)

3 The reason for $\frac{A_{i_{0}}}{A}|G_{A_{i_{0}}}(t)|$ appearing is that the supremum is attained by having $t_{i}=\frac{\pi }{2}$ for $i \ne i_{0}$ and hence $\prod _{i\ne i_{0}} G_{A_{i}}(0) = \prod _{i\ne i_{0}} 1/A_{i} = A_{i_{0}}/A$.
